# Supplementary material for: Amphibole reaction rims record shear during magma ascent
Source: Nat Commun. 2026 Apr 9;17:3407. doi: 10.1038/s41467-026-71477-x (PMC13068922; doi:10.1038/s41467-026-71477-x)
Supplement: Supplementary file 1 — Supplementary Information [file 41467_2026_71477_MOESM1_ESM.pdf]

Amphibole Reaction Rims Record Shear during Magma Ascent

Paul A. Wallace, Janine Birnbaum, Sarah H. De Angelis, Elisabetta Mariani, Jessica Larsen, Jackie E. Kendrick, Thomas E. Christopher, Paul D. Cole, Anthony Lamur, Yan Lavallée

Supplementary Information

Corresponding author: Paul A. Wallace (p.wallace@lmu.de)

Table of Contents

SAMPLE SELECTION AND REPRESENTATIVENESS **3**

Supplementary Fig. 1 3
Supplementary Table 1 4
Supplementary Table 2 5
Supplementary Fig. 2 6
Supplementary Fig. 3 7
Supplementary Fig. 4 8

AMPHIBOLE SETTLING MODEL **9**

Supplementary Fig. 5 9

NATURE OF TOPOTAXY FROM EBSD DATA **10**

EXAMPLE FROM SOUFRIERE HILLS VOLCANO **10**

Supplementary Fig. 6 10
Supplementary Fig. 7 11
Supplementary Fig. 8 12
Supplementary Fig. 9 13
Supplementary Fig. 10 14
Supplementary Fig. 11 15

EXAMPLE FROM EL MISTI **16**

Supplementary Fig. 12 16
Supplementary Fig. 13 17
Supplementary Fig. 14 18
Supplementary Fig. 15 19

EXTENDED METHODS FOR NUMERICAL MODELLING **20**

VELOCITY FIELDS AND INITIAL CONDITIONS . **20**

Supplementary Fig. 16 21
Supplementary Fig. 17 22
Supplementary Fig. 18 23
Supplementary Fig. 19 24

CALCULATION OF CRYSTAL ORIENTATIONS ... **25**

Supplementary Fig. 20 26
Supplementary Fig. 21 27

ASSEMBLING MIXED POPULATIONS OF CRYSTALS **27**

Supplementary Table 3 28

INVERSION OF NATURAL SAMPLES **29**

Supplementary Fig. 22 29
Supplementary Fig. 23 30

SUPPLEMENTARY REFERENCES **31**

Sample Selection and Representativeness

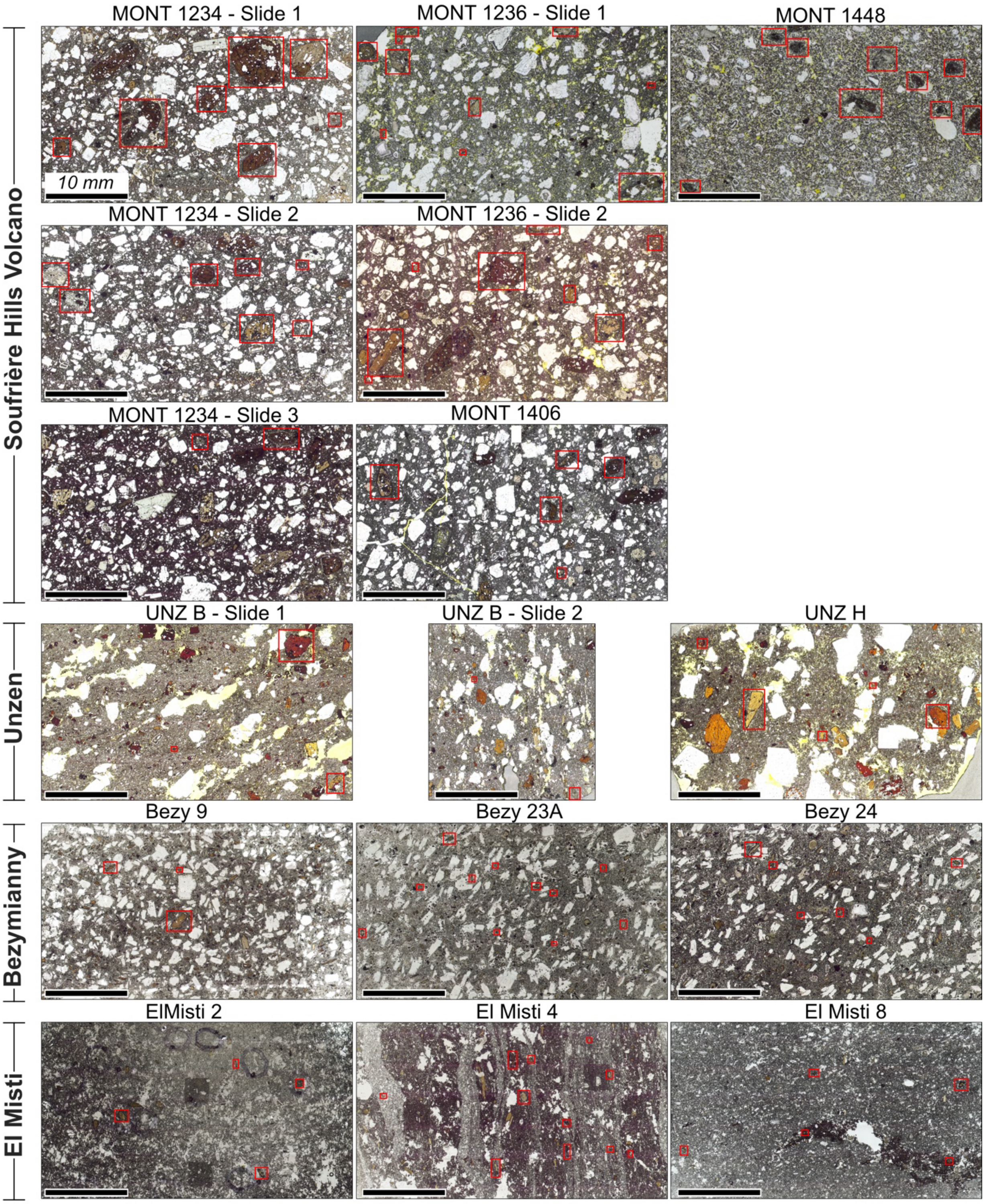

Supplementary Fig. 1: *Plane-polarized light (PPL) photomicrographs of the natural samples analysed from the four volcanic systems. Amphibole crystals selected for EBSD analysis are outlined in red. Amphiboles were chosen based on the presence of clearly developed reaction rims, while ensuring representativeness by targeting a range of rim thicknesses from thin to thick within each sample. All scale bars are 10 mm.*

Supplementary Table 1: *Natural amphibole-bearing samples used in this study, including sample context and number of amphibole rims analysed.*

SAMPLE NAME	SOURCE VOLCANO	SAMPLE DESCRIPTION	DATE OF ERUPTION	AMPHIBOLE RIMS ANALYSED	SAMPLE SOURCE	REFERENCE
**MONT 1234**	Soufrière Hills Volcano, Montserrat	Crystal-rich andesite. Pyroclastic flow deposit from White's Bottom Ghaut, 1 km from sea.	29 Sep 2002	16	Thomas Christopher (Montserrat Volcano Observatory)	N/A
**MONT 1236**	Soufrière Hills Volcano, Montserrat	Crystal-rich andesite. Pyroclastic flow and surge deposit.	12 Jul 2003	20	Thomas Christopher (Montserrat Volcano Observatory)	N/A
**MONT 1406**	Soufrière Hills Volcano, Montserrat	Dense jagged block, pale grey and porphyritic. Collected from Whites.	20 May 2006	5	Thomas Christopher (Montserrat Volcano Observatory)	N/A
**MONT 1448**	Soufrière Hills Volcano, Montserrat	Mafic inclusion from a pyroclastic flow deposit (dome rock) collected from Tar River Valley. Fine-grained matrix and crystals of amphibole and plagioclase (likely xenocrystic).	30 Jun 2006	9	Thomas Christopher (Montserrat Volcano Observatory)	N/A
**Bezy 9** (O6IPE9)	Bezymianny, Kamchatka	Andesitic directed blast deposit (lat/long: 55.92/160.69).	30 Mar 1956	3	Pavel Izbekov (UAF/AVO)	Ref. [1]
**Bezy 23A** (O5IPE23A)	Bezymianny, Kamchatka	Andesitic directed blast deposit (lat/long: 55.93/160.78).	30 Mar 1956	11	Pavel Izbekov (UAF/AVO)	Ref. [1]
**Bezy 24** (O5IPE24)	Bezymianny, Kamchatka	Pyroclastic flow deposit (lat/long: 55.93/160.78).	30 Mar 1956	6	Pavel Izbekov (UAF/AVO)	Ref. [1]
**ElMisti 2**	El Misti, Peru	Pumice from pyroclastic density current deposit (ignimbrite facies) at Quebada Grande (16°20'47.97"S, 71°22'46.55"W).	2000 B.P.	4	Shanaka de Silva (Oregon State University)	Ref. [2]
**ElMisti 4**	El Misti, Peru	Pumice from pyroclastic density current deposit (ignimbrite facies) at Quebada Grande (16°20'47.97"S, 71°22'46.55"W).	2000 B.P.	11	Shanaka de Silva (Oregon State University)	Ref. [2]
**ElMisti 8**	El Misti, Peru	Pumice from pyroclastic density current deposit (ignimbrite facies) at Quebada Grande (16°20'47.97"S, 71°22'46.55"W).	2000 B.P.	5	Shanaka de Silva (Oregon State University)	Ref. [2]
**UNZ-B**	Unzen, Japan	Dacite lava spine from the summit of Unzen lava dome (lat/long: 32.761313/130.2998260).	1995	5	Paul A. Wallace	Ref. [3]
**UNZ-H**	Unzen, Japan	Dacite lava spine from the summit of Unzen lava dome (lat/long: 32.761313/130.2998260).	1995	5	Paul A. Wallace	Ref. [3]

***Supplementary Table 2**: Summary of experimental conditions and amphibole rim analyses.*

SAMPLE NAME	STARTING MATERIAL	EXPERIMENTAL CONDITIONS*					AMPHIBOLE RIMS ANALYSED	REFERENCE
		T (ºC)	P (MPa)	Time (h)	fO_2	XCO_2		
**HSAS 31**	Finely ground fused glass derived from high-silica andesite pumice (06AUMC004c, Augustine Volcano, Alaska, 2006), seeded with hornblende crystals from Soufrière Hills Volcano, Montserrat, 2008 (SH08).	880	140	3	Re–ReO (~NNO+2)	0.0	4	Ref. [4]
**HSAS 03**	Same as above	900	140	3	Re–ReO (~NNO+2)	0.0	4	Ref. [4]
**HSAS 16**	Same as above	900	140	12	Re–ReO (~NNO+2)	0.0	4	Ref. [4]
**HSAS 17**	Same as above	900	140	24	Re–ReO (~NNO+2)	0.0	2	Ref. [4]
**HSAS 11**	Same as above	900	140	36	Re–ReO (~NNO+2)	0.0	1	Ref. [4]
**HSAS 06**	Same as above	900	140	48	Re–ReO (~NNO+2)	0.0	1	Ref. [4]
**HSAS 22**	Same as above	920	140	12	Re–ReO (~NNO+2)	0.0	6	Ref. [4]
**HSAS 08**	Same as above	920	140	36	Re–ReO (~NNO+2)	0.0	1	Ref. [4]
**RL_4**	Finely crushed crystal-poor rhyolite from Panum Crater, California, seeded with hornblende crystals from Soufrière Hills Volcano, Montserrat, 2008 (SH08).	880	120	24	NNO+1	0.3	9	Ref. [5]

* All samples were first equilibrated at 870 ºC and 140 MPa for 24 h, except for RL_4, which was first equilibrated at 830 ºC and 120 MPa for 24 h

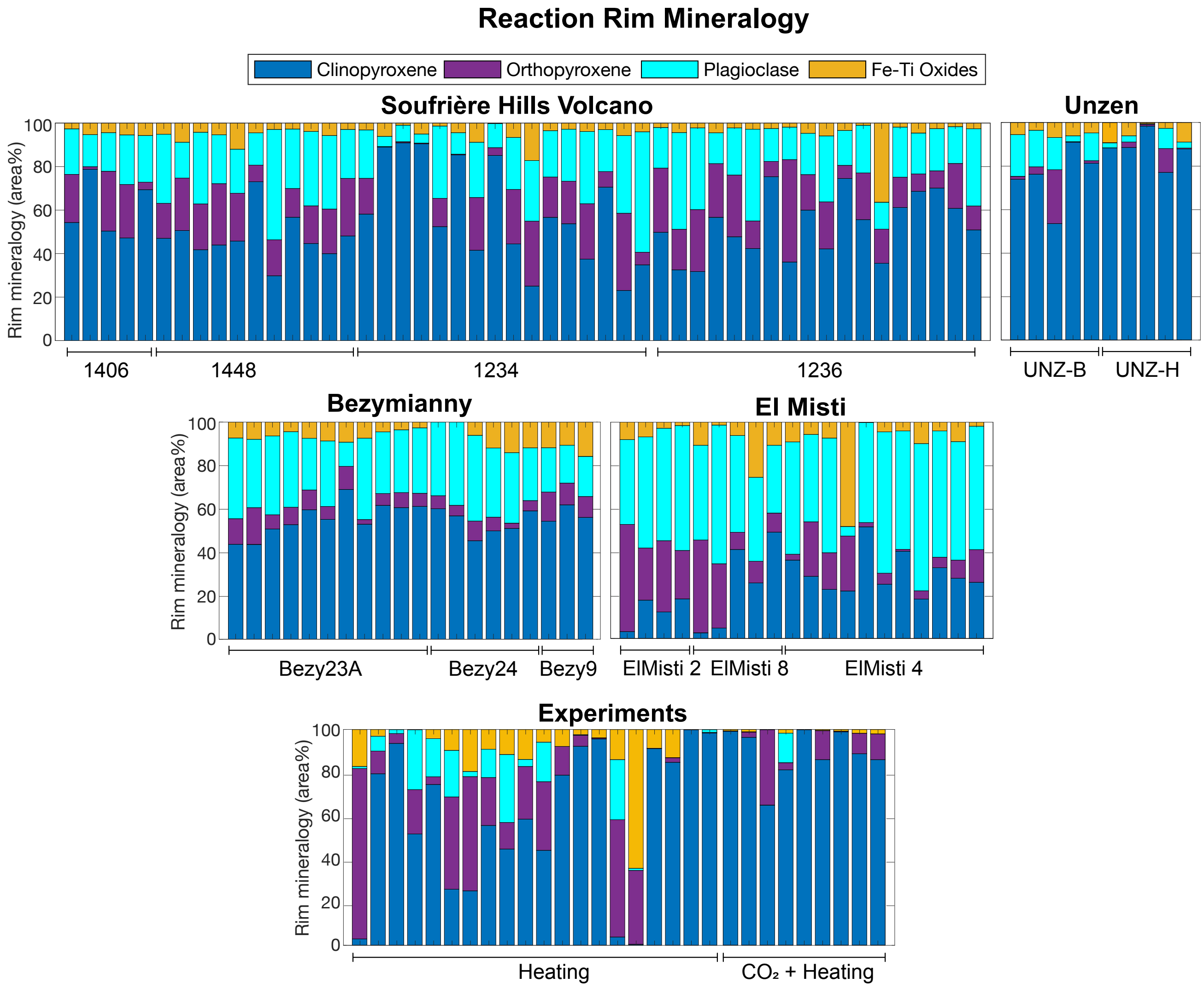

Supplementary Fig. 2*: Amphibole reaction rim mineral modal abundance for all natural and experimental amphibole reaction rims analysed in this study.*

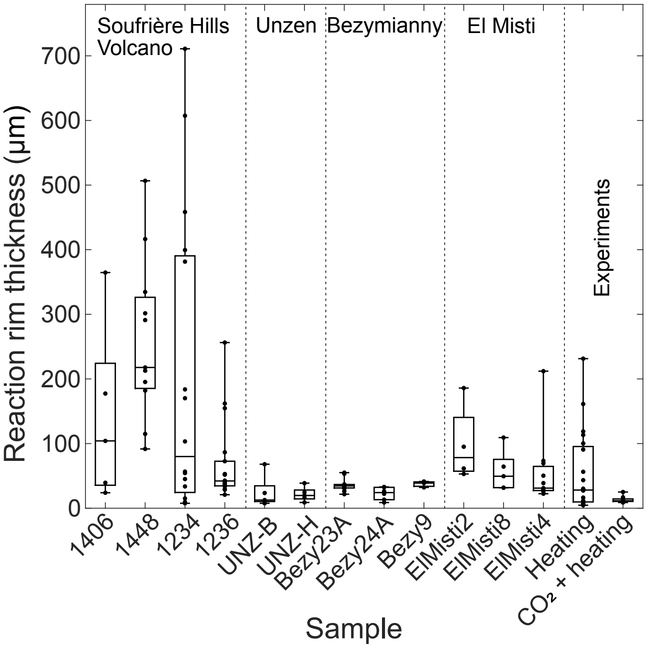

***Supplementary Fig. 3**: Reaction rim thickness of EBSD-targeted amphiboles by sample. Box-and-whisker plots show reaction rim thickness (µm) measured around each amphibole grain targeted for EBSD, taken as an average of five individual measurements around the amphibole. Boxes represent the interquartile range (25th–75th percentiles) with the median shown as a horizontal line. Whiskers extend to the minimum and maximum values (all measurements included; no outliers excluded). Individual amphibole measurements are overlaid as points to illustrate the full distribution.*

EBSD relative misorientation distributions

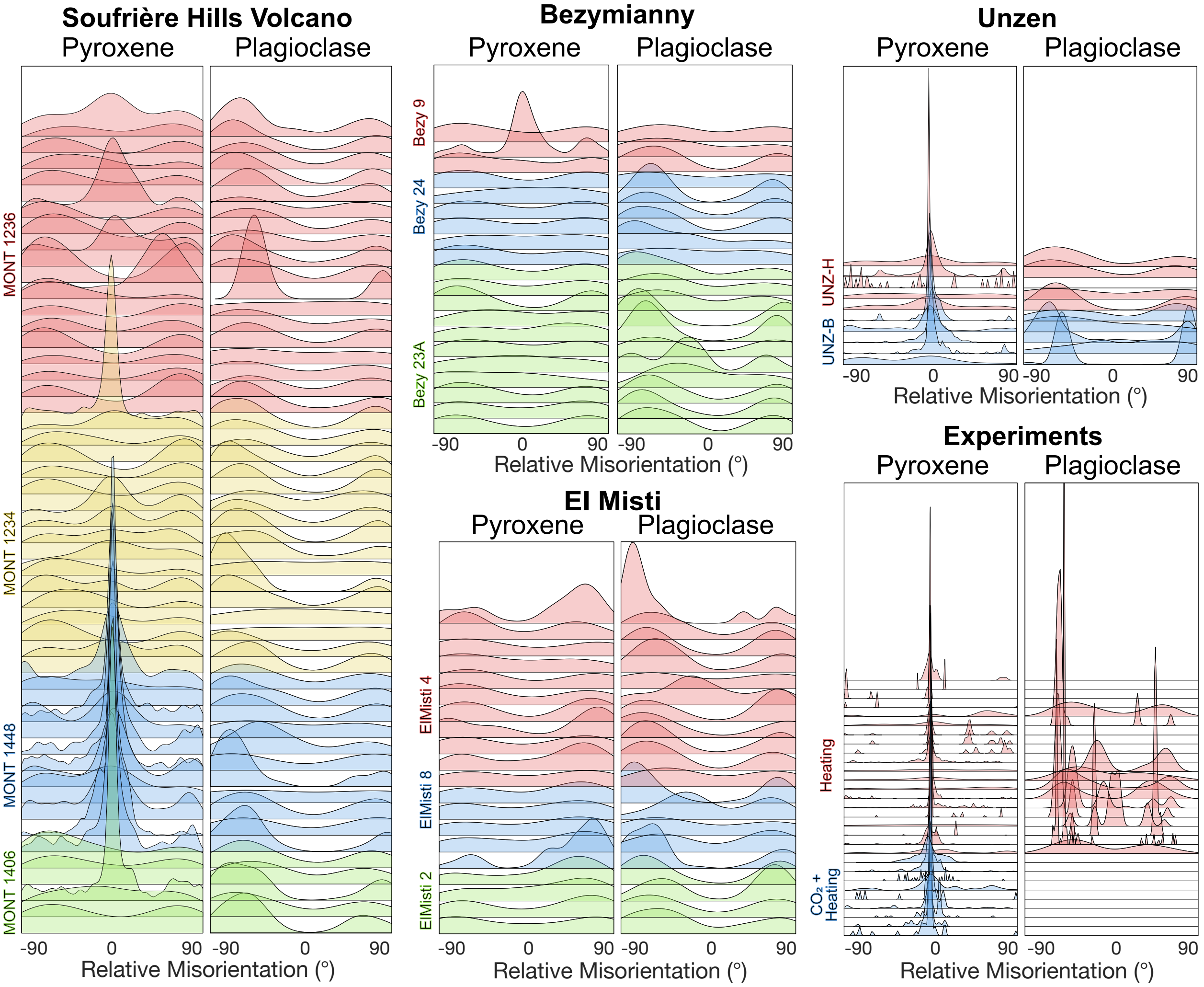

Supplementary Fig. 4: *Relative misorientation distributions for pyroxenes (combined clinopyroxene and orthopyroxene) and plagioclase present in all natural and experimental reaction rims, plotted as stacked kernel density estimates (KDEs). Each KDE represents a single amphibole reaction rim, with each colour (green, blue, yellow, red) corresponding to a single thin section/sample.*

Amphibole settling model

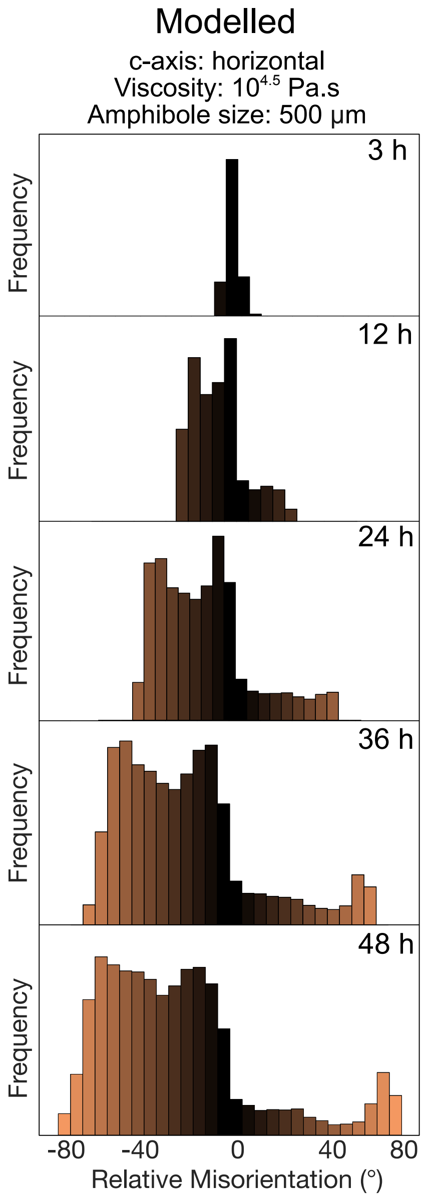

Supplementary Fig. 5*: Forward modelling of pyroxene misorientation distributions for an amphibole settling through time with its c-axis horizontal (see Fig. 3h for c-axis vertical).*

Nature of Topotaxy from EBSD data

To demonstrate the true crystallographic nature of topotaxy in amphibole reaction rims, we show detailed analyses of two representative examples: one from Soufrière Hills Volcano (sample MONT 1406) and one from El Misti Volcano (sample El Misti 4). These amphibole reaction rims were selected to illustrate contrasting misorientation relationships, one with well-preserved topotaxy (Soufrière Hills Volcano), and one with more dispersed orientation patterns (El Misti), allowing assessment of orientation inheritance and how deformation during amphibole breakdown alters evidence of topotaxy.

Example from Soufriere Hills Volcano

The example amphibole (hornblende; Supplementary Fig. 6a) from Soufrière Hills Volcano consists of a thick (~300–400 µm) reaction rim composed of clinopyroxene (augite), orthopyroxene (enstatite), plagioclase (anorthite) and Fe-Ti oxides (ilmenite and magnetite). The reaction rim is texturally zoned and can be separate into three discrete domains: (1) pyroxene inclusions inside the amphibole, (2) a fine-grained inner rim ~50–70 µm thick, and (3) a coarse-grained outer rim ~250–300 µm thick. The inverse pole figure (IPF) map of the Z direction (Supplementary Fig. 6b) illustrates the crystallographic orientation of each pixel within the amphibole grain and its surrounding rim phases relative to the sample surface normal, with colours corresponding to specific crystallographic directions along the Z-axis according to a defined colour key. This map reveals the crystal orientation of the hornblende grain and its rim phases, allowing visual identification of orientation domains, misorientations, and potential inheritance of crystallographic orientation across phase boundaries.

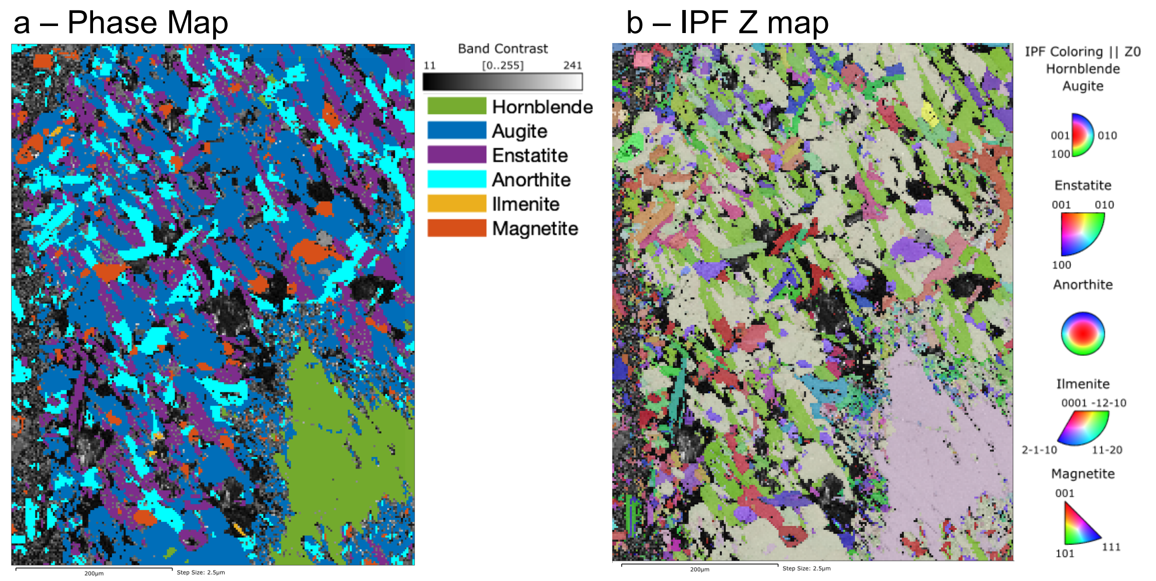

Supplementary Fig. 6*: (a) Phase map of a hornblende grain and its composite reaction rim from sample MONT 1406. (b) Inverse pole figure map of the Z direction in crystallographic coordinates. A colour key for all minerals analysed is shown on the right.*

The hornblende parent grain is undeformed and single-domain, as demonstrated by the uniform coloration in the all Euler map (Supplementary Fig. 7a) and the tightly clustered crystallographic axes in the upper hemisphere pole figures (Supplementary Fig. 7b). This consistent orientation confirms the hornblende grain maintains a coherent internal lattice, free from intracrystalline distortion, and thus provides a reliable crystallographic reference frame for evaluating the orientation of reaction rim phases. The associated crystal model (Supplementary Fig. 7c) illustrates the spatial orientation of the hornblende lattice relative to the sample coordinate system, enabling quantitative comparison of topotactic alignment in augite and enstatite across different rim zones.

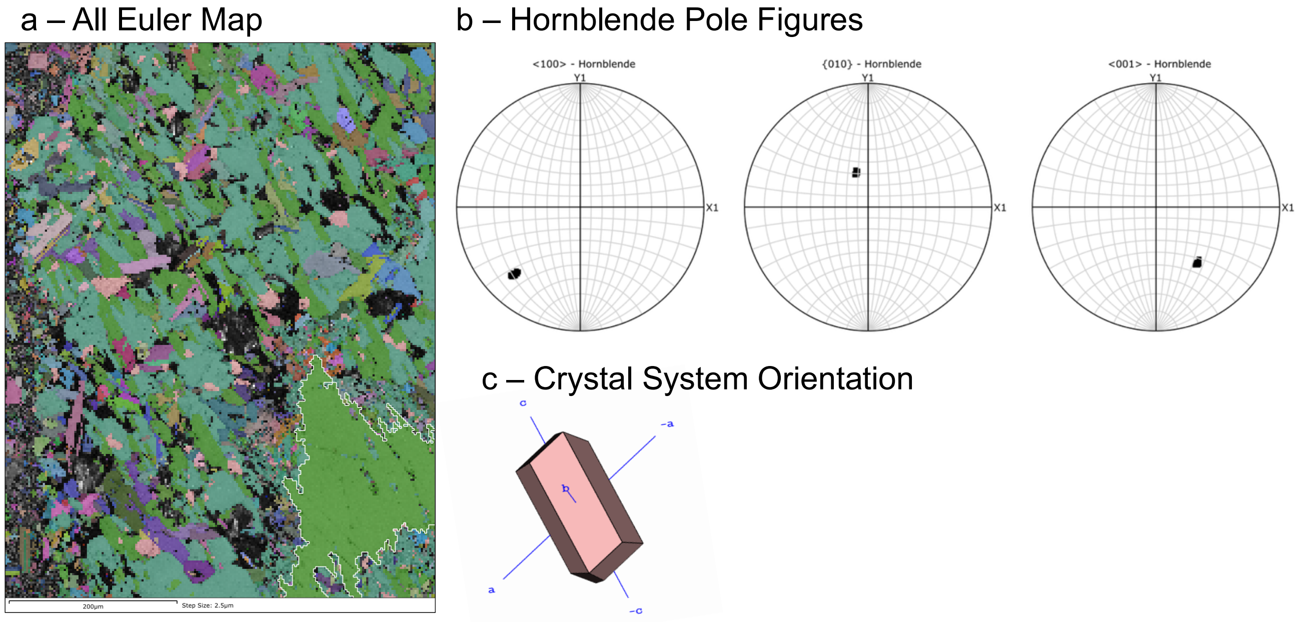

Supplementary Fig. 7*: (a) All Euler map of a hornblende grain (highlighted with a white outline) and its composite reaction rim from sample MONT 1406. (b) Equal angle, upper hemisphere pole figures showing the orientation of the crystallographic axes of the hornblende grain, plotted as one point per grain. This orientation is used as the reference orientation for the fabric of the hornblende reaction rim. (c) Amphibole crystal model demonstrating the orientation of the hornblende grain studied.*

Augite and enstatite occur as inclusions within the host hornblende grain (Supplementary Fig. 8a), providing a snapshot of early-stage amphibole breakdown. These inclusions display strong topotactic alignment with the amphibole, as shown by the concentrated crystallographic axes in the pole figures (Supplementary Fig. 8b–c). Enstatite inclusions grow with (100), (010), and [001] axes coincident with those of hornblende, indicating full 3D orientation inheritance. Augite inclusions also retain [001] parallel to hornblende but exhibit a 180° rotation about this axis, producing a mirror-like relationship across the (100) plane, a known twin plane in both augite and amphibole. Crystal models comparing the orientations of augite and enstatite inclusions to the hornblende reference (Supplementary Fig. 8d–f) demonstrate these relationships and highlight the systematic alignment of lattice directions, albeit with augite flipped 180°. Both augite [100] and [001] display small-angle great-circle rotations around [010], suggesting that [010] serves as the dominant rotation axis even during early stages of replacement.

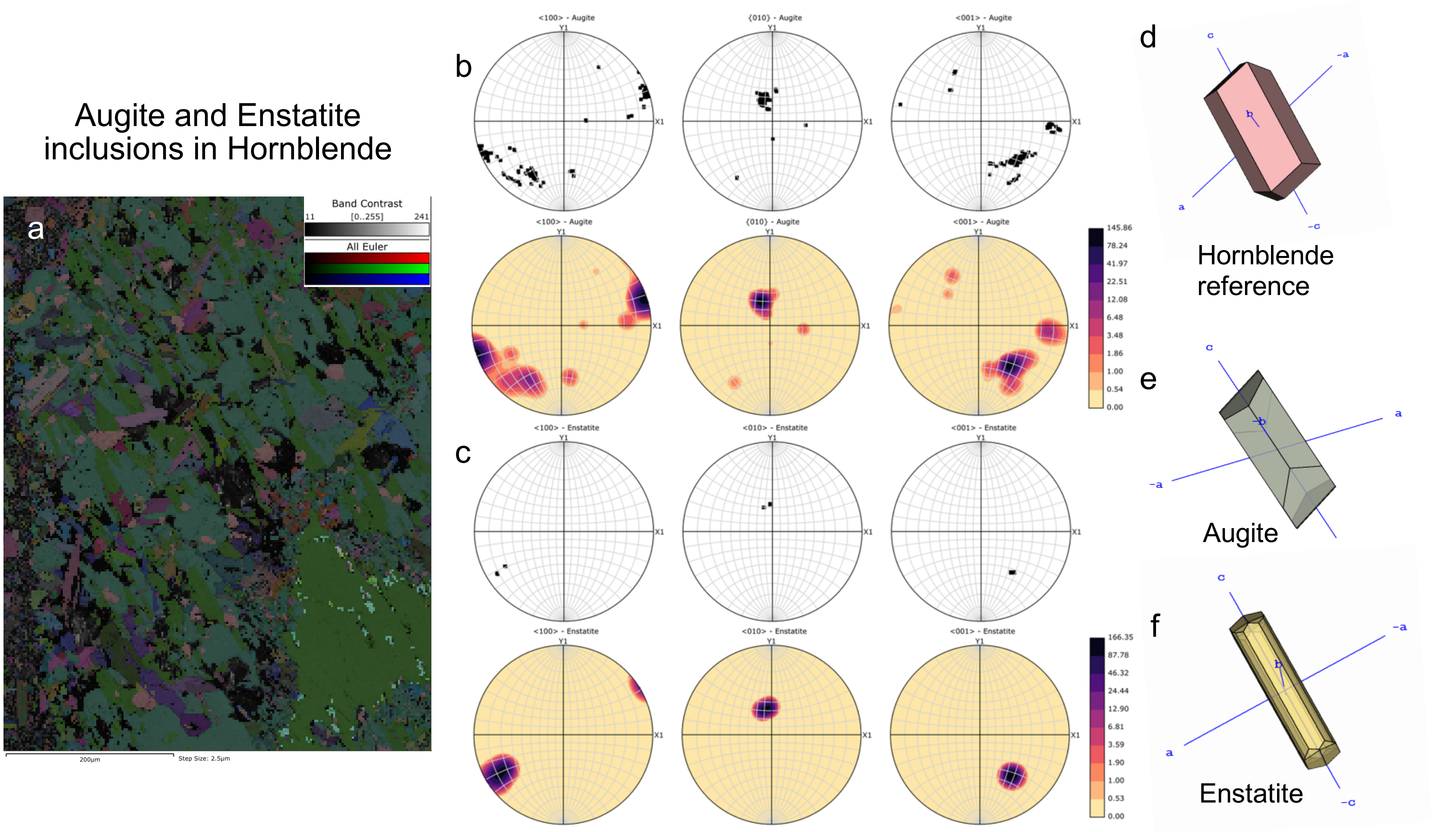

Supplementary Fig. 8*: a) All Euler map of hornblende and its composite reaction rim from sample MONT 1406. Augite and enstatite inclusions within hornblende are highlighted. b) Equal angle, upper hemisphere pole figures showing the orientation of the crystallographic axes of the augite inclusions, plotted as one point per grain. (c) Equal angle, upper hemisphere pole figures showing the orientation of the crystallographic axes of the enstatite inclusions, plotted as one point per grain. Colour bar for (b–c) represents Multiples of Uniform Distribution (MUD), which is a quantification of the degree of preferred crystallographic orientation (i.e., the strength of fabric or texture). (d–f) Crystal models comparing the crystallographic orientation of the reference hornblende grain with augite and enstatite inclusions.*

In the fine-grained inner rim, augite and enstatite grains display increased crystallographic dispersion, with intracrystalline misorientation reaching up to ~3º, which is relatively large for such small grains (Supplementary Fig. 9a). Pole figures for augite and enstatite (Supplementary Fig. 9b–c) show that both [100] and [001] axes undergo large-angle great-circle rotations around [010], indicating that [010] continues to act as the dominant rotation axis during amphibole breakdown. The pole figure clustering remains non-random and directionally consistent, suggesting that despite moderate deformation, pyroxene crystallisation is still strongly influenced by the hornblende orientation. These rotations possibly reflect syn-crystallisation reorientation driven by magma shear, where [001] is subparallel to the flow directions and both [001] and [010] are contained in the plane of flow. The presence of coherent patterns, despite increased dispersion, highlights the persistence of crystallographic control even in strain-affected portions of the rim.

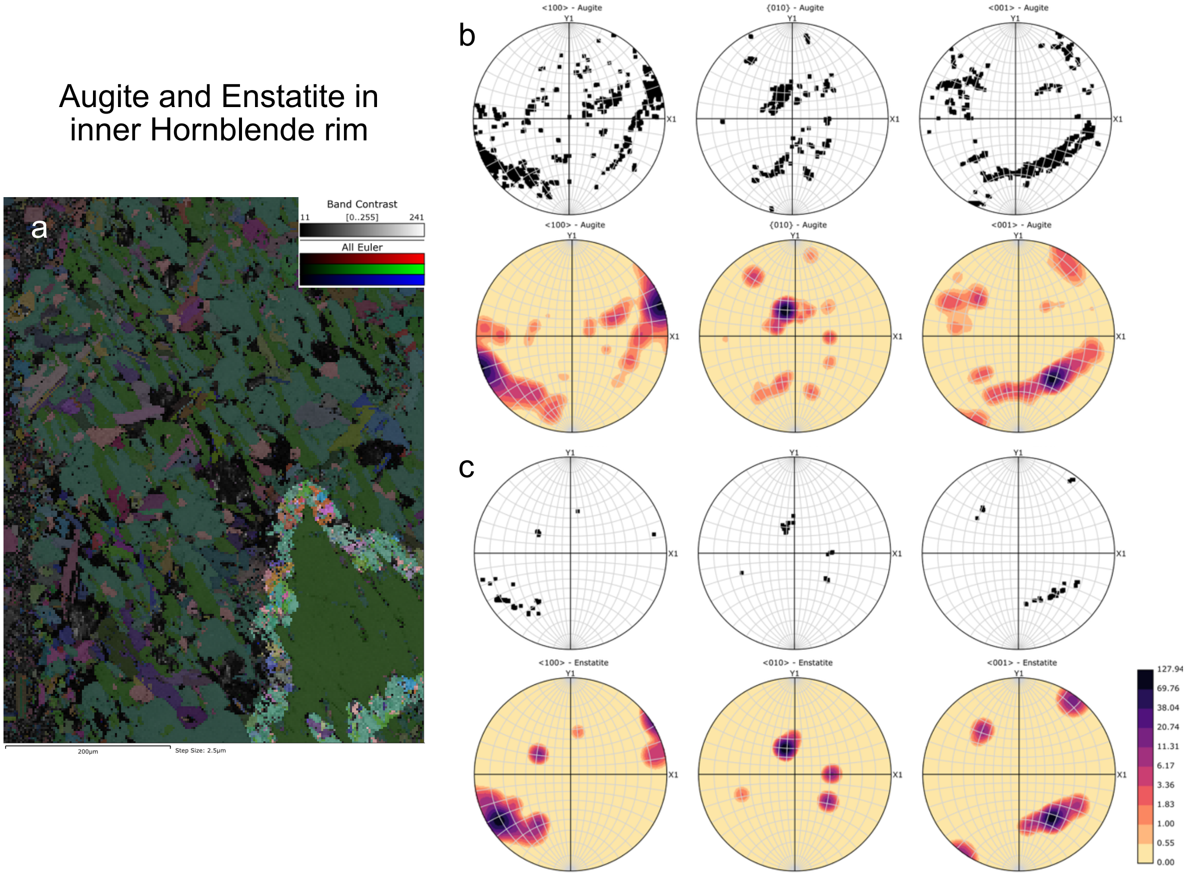

Supplementary Fig. 9*: (a) All Euler map of hornblende and its composite reaction rim from sample MONT 1406. Augite and enstatite grains from the finer grained inner part of the reaction rim are highlighted. (b) Equal angle, upper hemisphere pole figures showing the orientation of the crystallographic axes of the inner rim augite grains, plotted as one point per grain. (c) Equal angle, upper hemisphere pole figures showing the orientation of the crystallographic axes of the inner rim enstatite grains, plotted as one point per grain. Colour bar for (c)represents Multiples of Uniform Distribution (MUD), a quantification of the degree of preferred crystallographic orientation (i.e., the strength of fabric).*

In the coarser-grained outer rim, augite and enstatite grains exhibit lower intracrystalline distortion, with misorientations typically below 2º, indicating reduced deformation compared to the inner rim (Supplementary Fig. 10a). Pole figures for both augite and enstatite (Supplementary Fig. 10b–c) show that [100], [001], and [010] axes remain well-clustered and undergo only small-angle great-circle rotations, confirming strong preservation of the original topotactic relationship with the host hornblende. The limited spread in crystallographic axes and high multiples of uniform distribution (MUD) values further indicate that, despite being part of a reaction rim, these pyroxenes retained tight crystallographic coupling to the amphibole lattice.

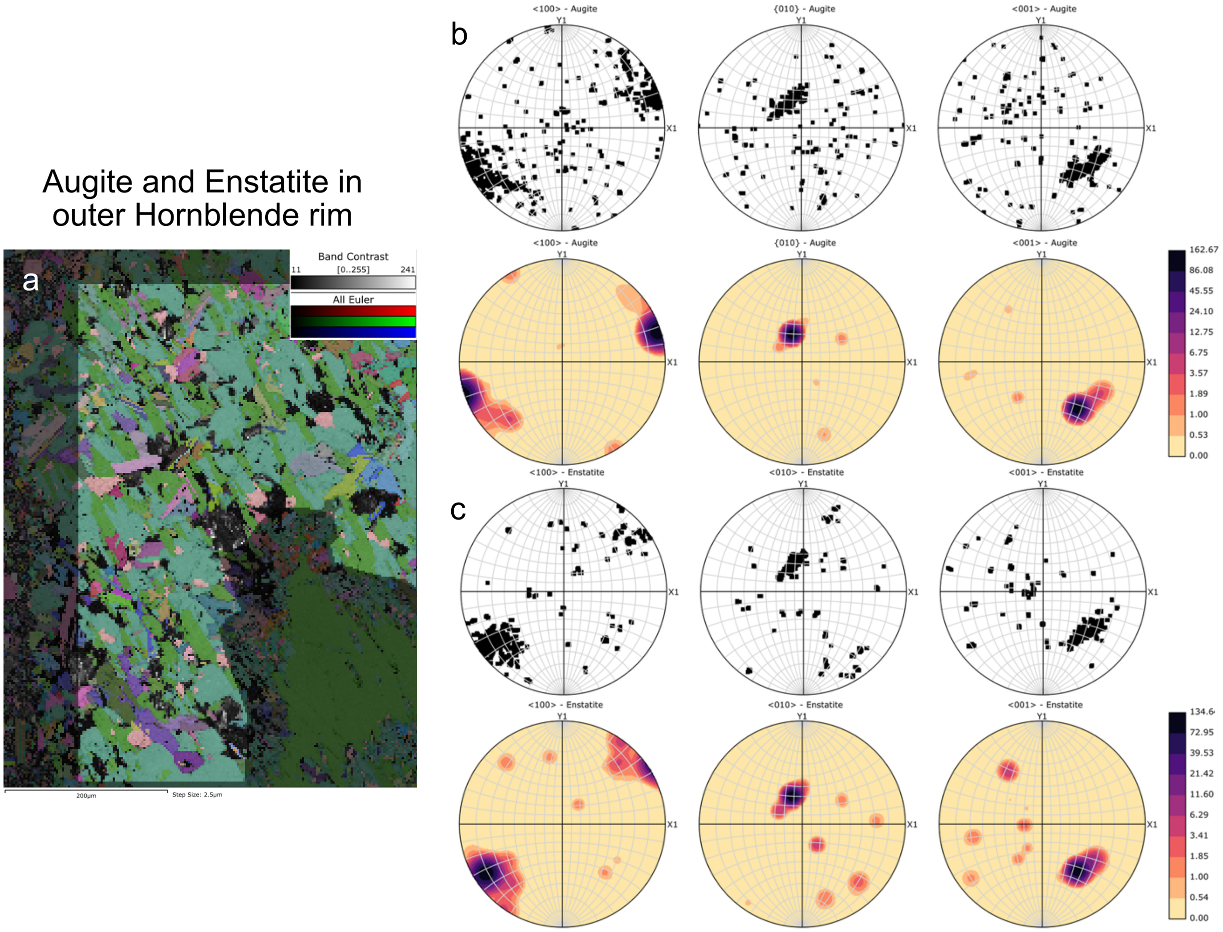

Supplementary Fig. 10*: (a) All Euler map of hornblende and its composite reaction rim from sample MONT 1406. Augite and enstatite grains from the coarser grained outer part of the reaction rim are highlighted. (b) Equal angle, upper hemisphere pole figures showing the orientation of the crystallographic axes of the outer rim augite grains, plotted as one point per grain. (c) Equal angle, upper hemisphere pole figures showing the orientation of the crystallographic axes of the outer rim enstatite grains, plotted as one point per grain. Colour bar for (b–c) represents Multiples of Uniform Distribution (MUD), which is a quantification of the degree of preferred crystallographic orientation (i.e., the strength of fabric or texture).*

Inverse pole figures (IPF) of the X, Y, and Z sample directions (Supplementary Fig. 11) confirm strong crystallographic alignment between the hornblende host (Supplementary Fig. 11b) and the outer rim pyroxenes (Supplementary Fig. 11c–d), with augite and enstatite grains showing closely matching orientation clusters relative to the hornblende reference frame. The IPFs reveal that the dominant crystallographic axes in augite and enstatite remain strongly aligned with those of hornblende across all principal sample directions, consistent with preserved topotactic inheritance. Notably, the data show that (100) continues to serve as a plane of symmetry (or mirror plane) between hornblende and augite, reflecting the 180º rotation about [001] that characterises the topotactic relationship in clinopyroxene.

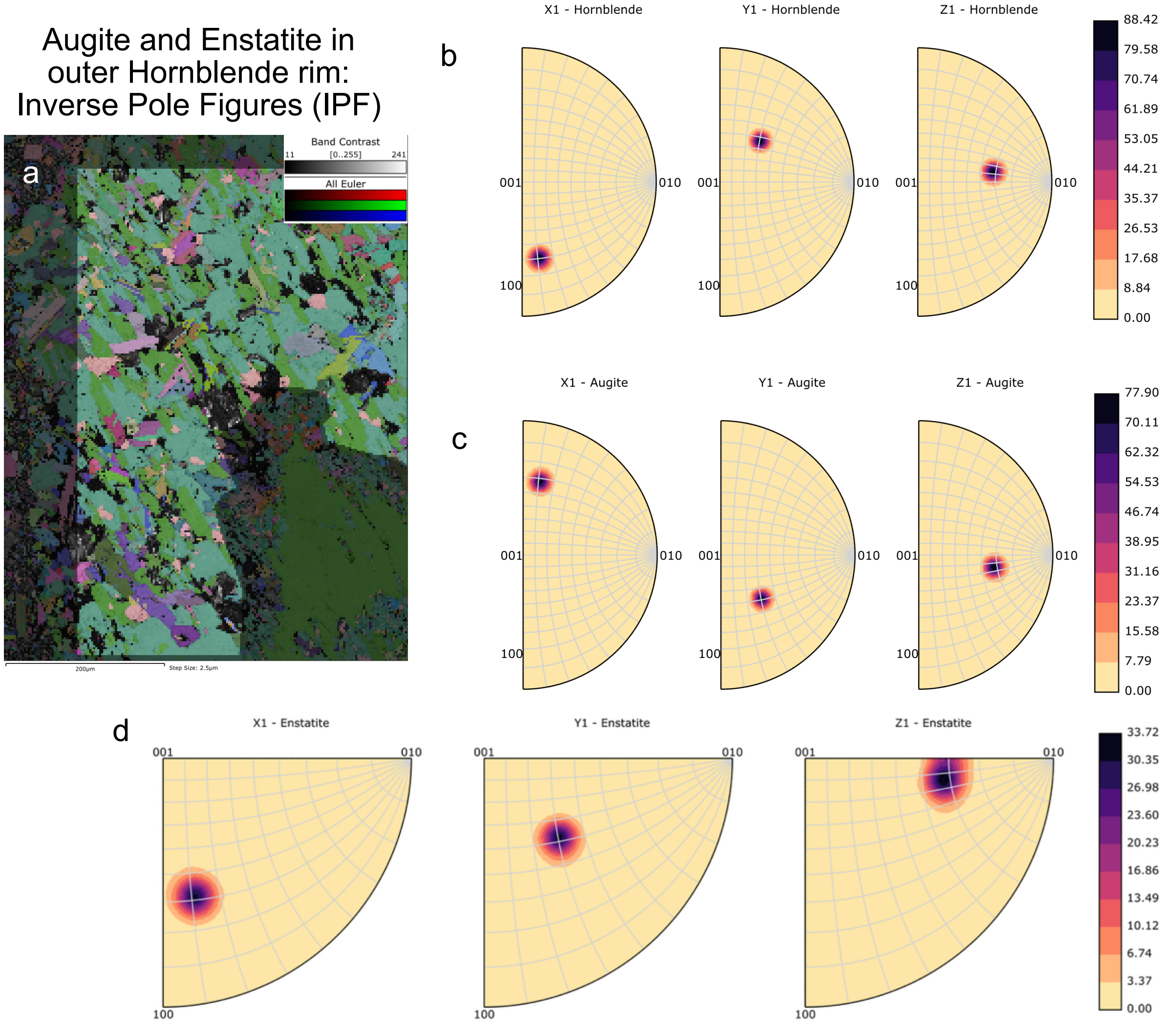

***Supplementary Fig. 11**: (a) All Euler map of hornblende and its composite reaction rim from sample MONT 1406. Augite and enstatite grains from the coarser grained outer part of the reaction rim are highlighted. (b) Inverse Pole Figures (IPF) showing the orientation of the crystallographic axes of the hornblende grains, plotted as one point per grain. (c) IPF showing the orientation of the crystallographic axes of the outer rim augite grains, plotted as one point per grain. (c) IPF showing the orientation of the crystallographic axes of the outer rim enstatite grains, plotted as one point per grain. Colour bar for (b–d) represents Multiples of Uniform Distribution (MUD), which is a quantification of the degree of preferred crystallographic orientation (i.e., the strength of fabric or texture).*

Example from El Misti

The example amphibole (hornblende; Supplementary Fig. 12a) from El Misti consists of a ~30–50 μm thick reaction rim composed of clinopyroxene (augite), orthopyroxene (enstatite), plagioclase (anorthite) and Fe-Ti oxides (ilmenite and magnetite). Unlike the example from Soufrière Hills Volcano, the reaction rim is texturally coherent. The IPF map of the Z direction (Supplementary Fig. 12b) illustrates the crystallographic orientation of each pixel within the amphibole grain and its surrounding rim phases relative to the sample surface normal, with colours corresponding to specific crystallographic directions along the Z-axis according to a defined colour key. In contrast to Soufrière Hills Volcano example, the El Misti sample (El Misti 4) shows weaker topotactic preservation between the host amphibole and reaction rim.

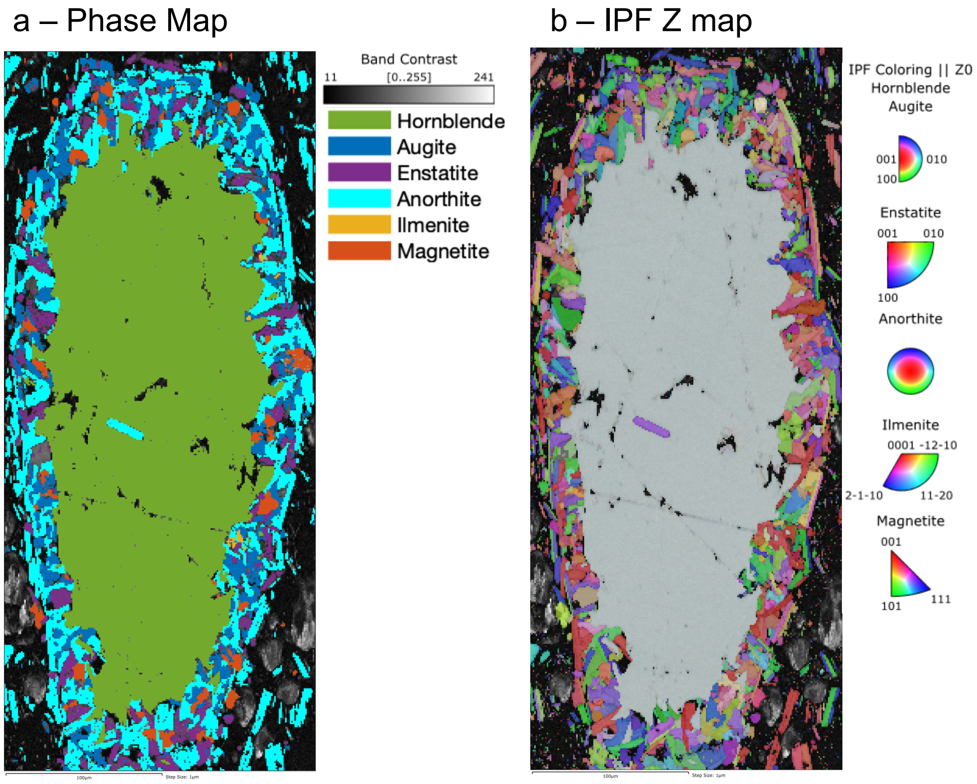

Supplementary Fig. 12*: A) Phase map of a hornblende grain and its composite reaction rim from sample El Misti 4. A) Inverse pole figure map of the Z direction in crystallographic coordinates. A colour key for all minerals analysed is shown on the right.*

The hornblende parent grain from El Misti is structurally intact and exhibits a single crystallographic orientation, as indicated by the homogeneous coloration in the all Euler map (Supplementary Fig. 13a) and the well-clustered crystal axes in the pole figures (Supplementary Fig. 13b). The absence of internal misorientation confirms that the amphibole grain is undeformed, making it a suitable reference for assessing the orientation of newly formed rim minerals. The accompanying crystal model (Supplementary Fig. 13c) defines the hornblende's lattice orientation within the sample coordinate system, allowing evaluation of crystallographic relationships between the amphibole and associated augite and enstatite in the surrounding reaction rim.

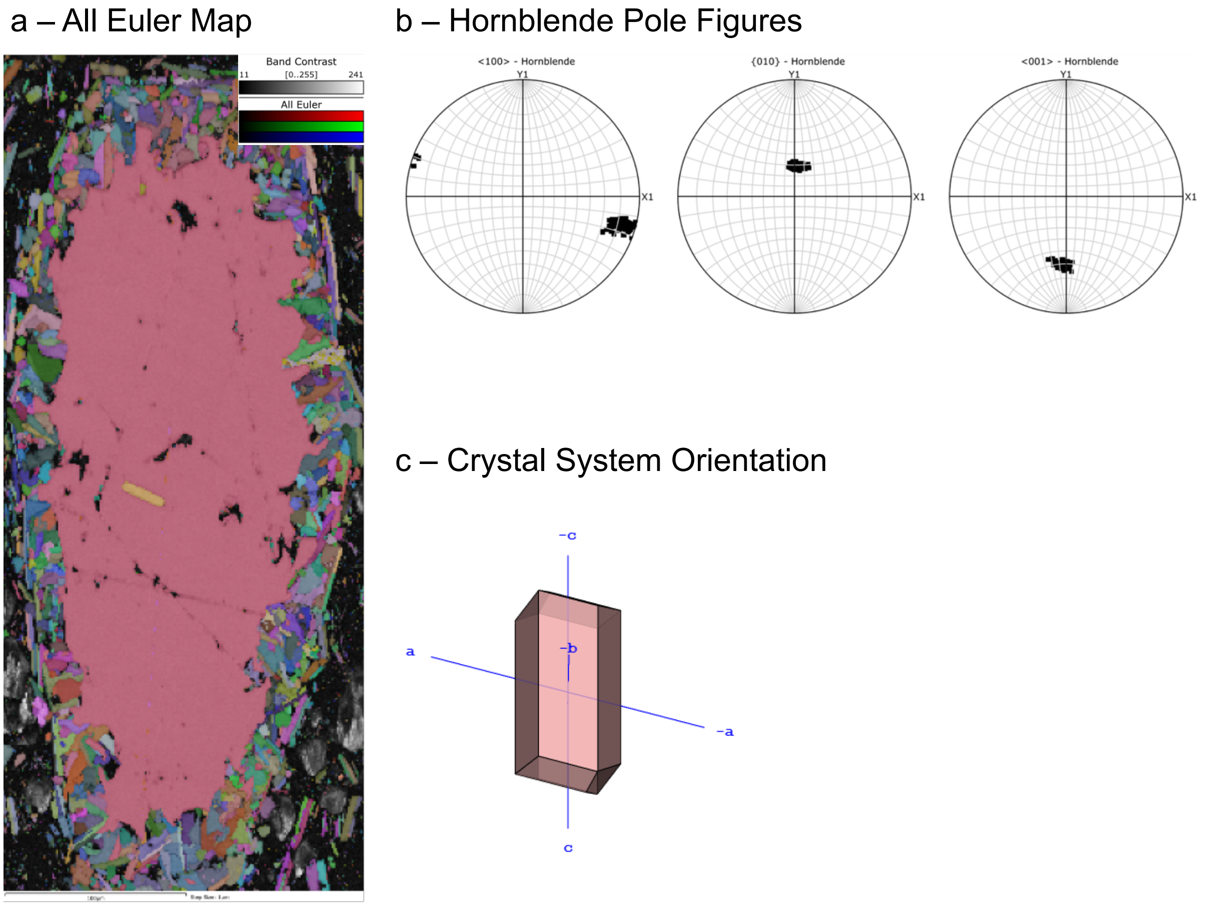

Supplementary Fig. 13*: (a) All Euler map of a hornblende grain (highlighted with a white outline) and its composite reaction rim from sample El Misti 4. (b) Equal angle, upper hemisphere pole figures showing the orientation of the crystallographic axes of the hornblende grain, plotted as one point per grain. This orientation is used as the reference orientation for the fabric of the hornblende reaction rim. (c) Amphibole crystal model demonstrating the orientation of the hornblende grain studied.*

In both augite and enstatite, poles figures reveal [100] axes cluster in the X1 direction of the hornblende, while [001] axes form a broad girdle within the YZ plane (Supplementary Fig. 14b–c). This configuration indicates that the main topotactic relationship is through (100) alignment, still mappable despite the overall weak fabric. [010] displays the least alignment, due to combined dispersion of [100] in a broad cluster and [001] in the YZ girdle. These rotations are interpreted as strain-induced, driven by magma flow around the amphibole grain, where augite and enstatite (100) are parallel to the flow plane and remain steady, while [001] are dispersed within the same plane.

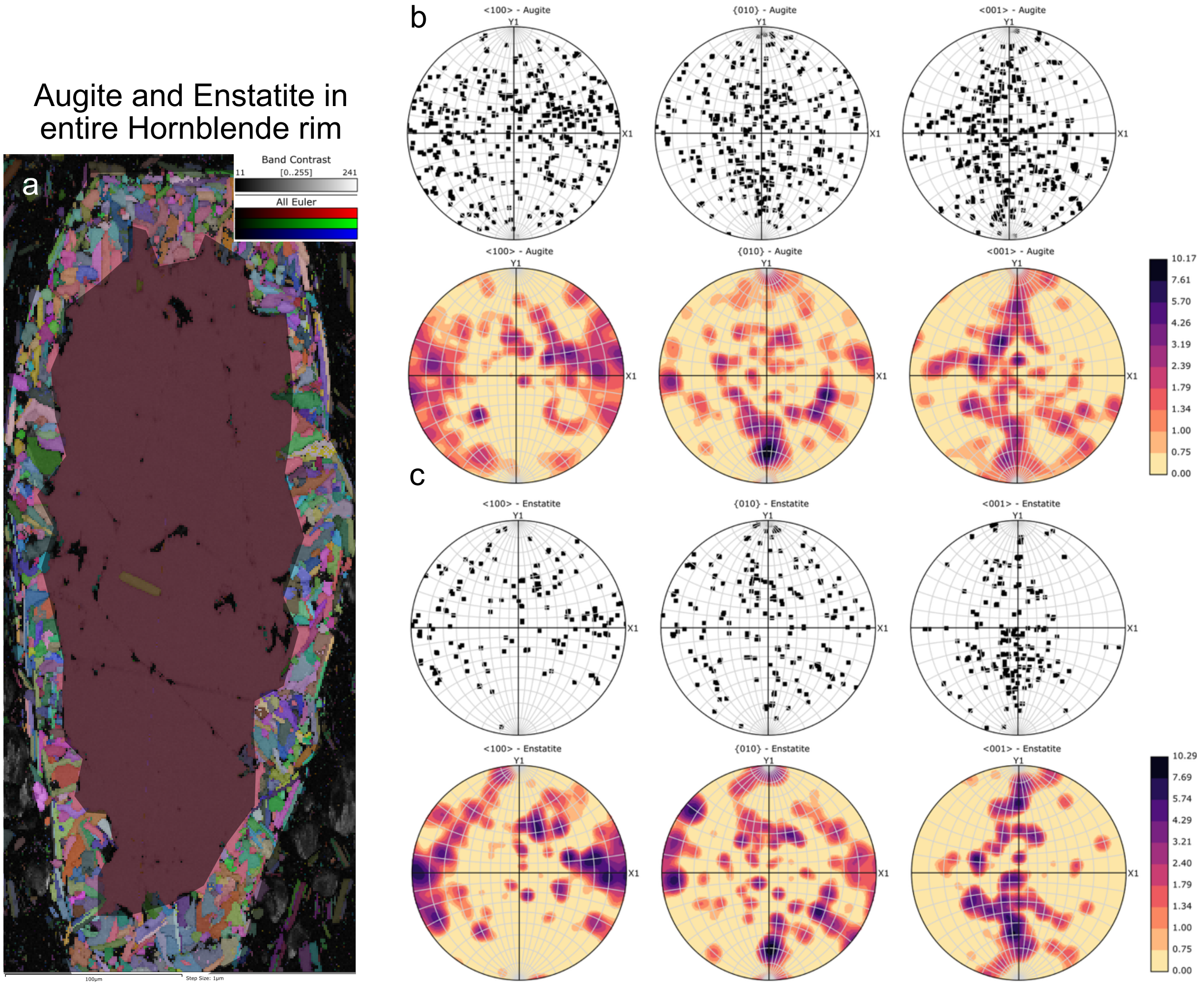

Supplementary Fig. 14*: (a) All Euler map of hornblende and its composite reaction rim from sample El Misti 4. Augite and enstatite grains from the reaction rim are highlighted. (b) Equal angle, upper hemisphere pole figures showing the orientation of the crystallographic axes of all augite grains in the reaction rim, plotted as one point per grain. (c) Equal angle, upper hemisphere pole figures showing the orientation of the crystallographic axes of all enstatite grains in the reaction rim, plotted as one point per grain. Colour bar for (b–c) represents Multiples of Uniform Distribution (MUD), which is a quantification of the degree of preferred crystallographic orientation (i.e., the strength of fabric or texture).*

Inverse pole figures (Supplementary Fig. 15) show that, although augite and enstatite grains in the El Misti reaction rim exhibit a broader spread in crystallographic orientations, their distributions are not random. Both pyroxene phases display preferred alignment patterns (Supplementary Fig. 15c–d), particularly in the orientation of [100], which remain clustered relative to the hornblende reference frame (Supplementary Fig. 15b). This confirms that, despite a weaker overall fabric, the orientation of the rim pyroxenes remains crystallographically constrained and demonstrates that the initial topotactic inheritance from the amphibole is still recognisable. The high MUD values further support the presence of a low-strength crystallographic fabric, indicating that deformation has broadened but not destroyed the underlying topotactic signal.

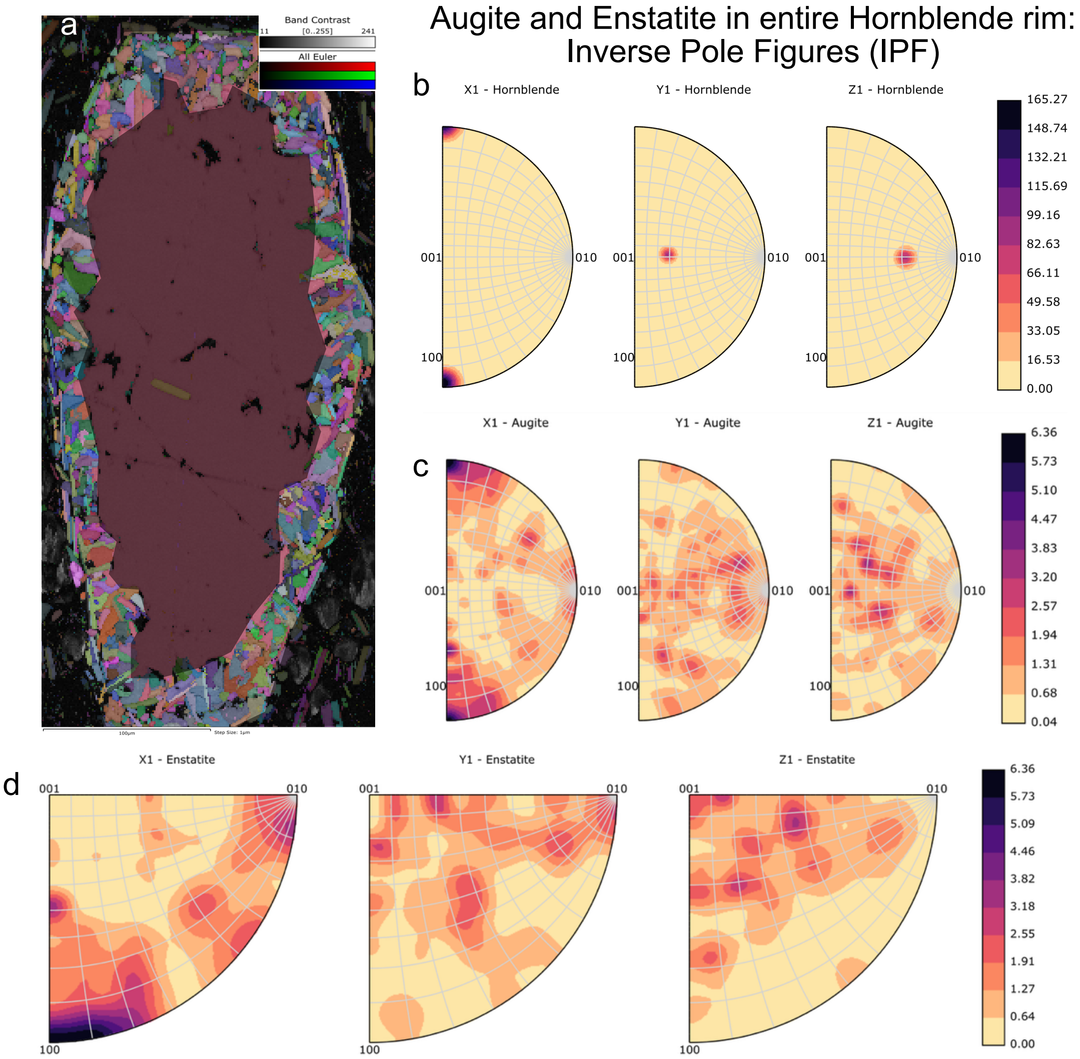

***Supplementary Fig. 15**: (a) All Euler map of hornblende and its composite reaction rim from sample El Misti 4. Augite and enstatite grains from the reaction rim are highlighted. (b) Inverse Pole Figures (IPF) showing the orientation of the crystallographic axes of the hornblende grains, plotted as one point per grain. (c) IPF showing the orientation of the crystallographic axes of augite grains in the reaction rim, plotted as one point per grain. (c) IPF showing the orientation of the crystallographic axes of all enstatite grains in the reaction rim, plotted as one point per grain. Colour bar for (b–d) represents Multiples of Uniform Distribution (MUD), a quantification of the degree of preferred crystallographic orientation (i.e., the strength of fabric).*

Extended Methods for Numerical Modelling

Velocity fields and initial conditions

Gravitational settling

The gravitational settling of a sphere in an iso-viscous liquid was first solved by Stokes[6] under the conditions of negligible Reynolds number, incompressibility (Stokes assumptions), no far-field stress, and no-slip along the surface of the sphere. For convenience the problem is typically solved in axisymmetric cylindrical coordinates with the rotational axis of symmetry aligned with the direction of gravity centered at the center of the sphere in a frame of reference which follows the sphere. The result is

$$u_r = \frac{3Arzu_{settling}}{4\sqrt{r^2+z^2}}\left(\left(\frac{A}{r^2+z^2}\right)^2 - \frac{1}{r^2+z^2}\right) \tag{1}$$

$$u_z = u_{settling} + \frac{3Au_{settling}}{4\sqrt{r^2+z^2}}\left(\frac{2A^2+3r^2}{3(r^2+z^2)} - \left(\frac{rA}{r^2+z^2}\right)^2 - 2\right) \tag{2}$$

for the radial coordinate r, vertical coordinate z, sphere radius A, and settling velocity $u_{settling}$. For

$$u_{settling} = \frac{2\Delta\rho g A^2}{9\mu} \tag{3}$$

given a density contrast $\Delta\rho$, gravitational acceleration g, and fluid viscosity μ. We neglect the individual settling of the clinopyroxene crystals on account of their much smaller effective radius (<1/20 A).

In cartesian coordinates:

$$u_x = \cos\left(\tan^{-1}\frac{y}{x}\right)\frac{3A\sqrt{x^2+y^2}zu_{settling}}{4\sqrt{x^2+y^2+z^2}}\left(\left(\frac{A}{x^2+y^2+z^2}\right)^2 - \frac{1}{x^2+y^2+z^2}\right) \tag{4}$$

$$u_y = \sin\left(\tan^{-1}\frac{y}{x}\right)\frac{3A\sqrt{x^2+y^2}zu_{settling}}{4\sqrt{x^2+y^2+z^2}}\left(\left(\frac{A}{x^2+y^2+z^2}\right)^2 - \frac{1}{x^2+y^2+z^2}\right) \tag{5}$$

$$u_z = u_{settling} + \frac{3Au_{settling}}{4\sqrt{x^2+y^2+z^2}}\left(\frac{2A^2+3(x^2+y^2)}{3(x^2+y^2+z^2)} - \left(\frac{\sqrt{x^2+y^2}A}{x^2+y^2+z^2}\right)^2 - 2\right) \tag{6}$$

For which we can analytically determine the derivatives that constitute the strain rate matrix:

$$\dot{\epsilon} = \begin{bmatrix} \sigma_{xx} & \tau_{xy} & \tau_{xz} \\ \tau_{xy} & \sigma_{yy} & \tau_{yz} \\ \tau_{xz} & \tau_{yz} & zz \end{bmatrix} = \begin{bmatrix} \frac{\partial u_x}{\partial x} & \frac{1}{2}\left(\frac{\partial u_x}{\partial y} + \frac{\partial u_y}{\partial x}\right) & \frac{1}{2}\left(\frac{\partial u_x}{\partial z} + \frac{\partial u_z}{\partial x}\right) \\ \frac{1}{2}\left(\frac{\partial u_x}{\partial y} + \frac{\partial u_y}{\partial x}\right) & \frac{\partial u_y}{\partial y} & \frac{1}{2}\left(\frac{\partial u_y}{\partial z} + \frac{\partial u_z}{\partial y}\right) \\ \frac{1}{2}\left(\frac{\partial u_x}{\partial z} + \frac{\partial u_z}{\partial x}\right) & \frac{1}{2}\left(\frac{\partial u_y}{\partial z} + \frac{\partial u_z}{\partial y}\right) & \frac{\partial u_z}{\partial z} \end{bmatrix} \quad (7)$$

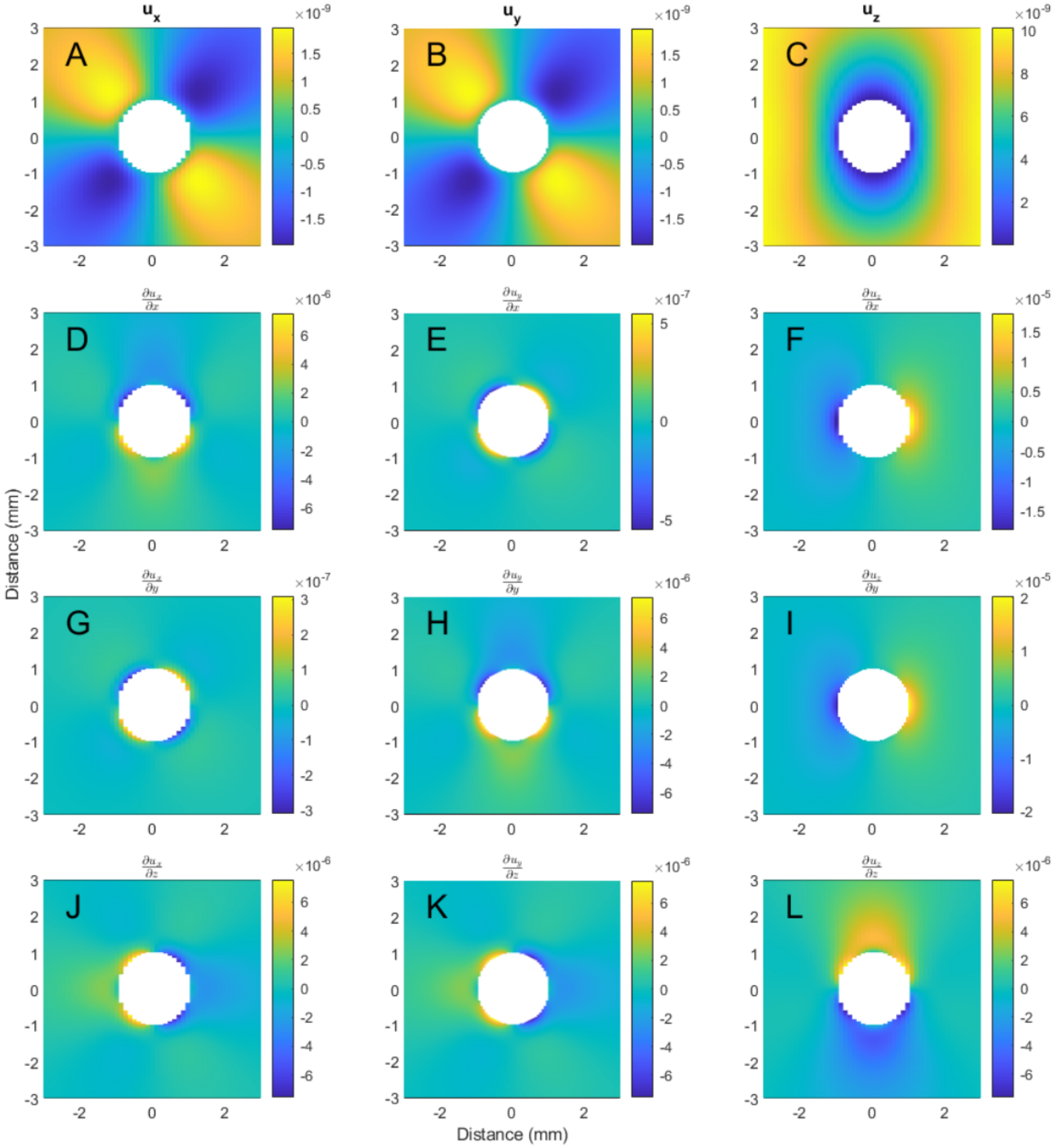

Supplementary Fig. 16*: Analytical solutions for velocity (A-C) and strain rates (B-L) surrounding a falling sphere with a diameter of 1 mm and a settling velocity of 6.85* $\times 10^{-9}$ *m/s, consistent with a density contrast of 200 kg/m*^{3} *and a fluid viscosity of 5×10*^{4} *Pa·s and a gravitational acceleration of 9.81 m/s*^{2}*.*

5×10^4 clinopyroxenes were initialized with uniformly random radial distances 1-1.03A, uniformly random azimuth in the xy-plane, and a normal distribution in inclination with respect to the z-axis centered about horizontal to produce relatively even spatial coverage around the sphere's surface.

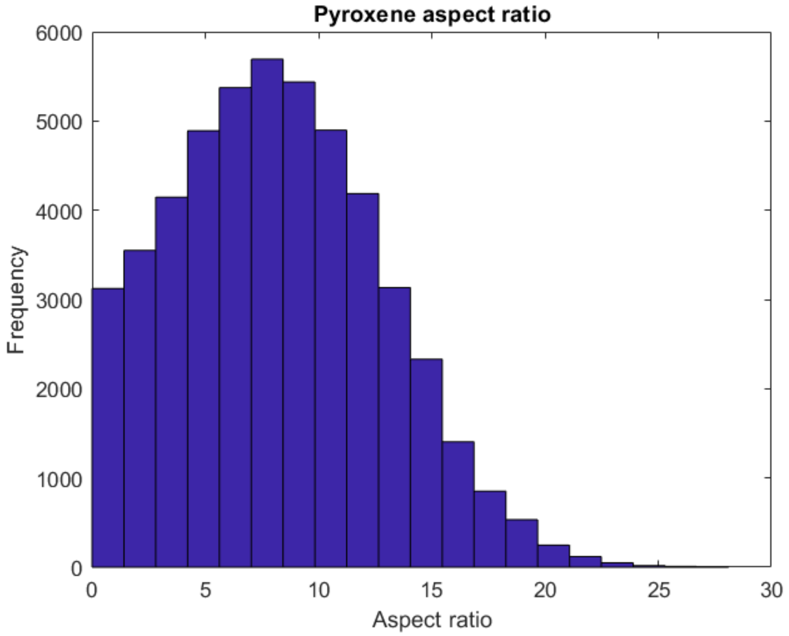

Supplementary Fig. 17*: Histogram of aspect ratios for clinopyroxenes.*

Lid-driven cavity flow

To match a wide crack-like geometry that is observed in the samples, we simplify the flow pattern to a modification of the lid-driven cavity problem which consists of an upper boundary of uniform velocity in the horizontal direction and no-slip conditions along the cavity walls. The circulation flow driven in the cavity lacks an analytical solution for the generic case, and indeed has singularities at the upper corners, but nonetheless is commonly used to verify numerical solutions for fluid flow and has been explored in a wide range of conditions. We maintain the simplifications of iso-viscosity and low Reynold's number with no-slip conditions within the crack. We choose a characteristic crack separation and depth of A with parallel walls. Because we desire no-slip conditions along the outer surface of the amphibole in addition to the crack walls, we extend the domain to a height of A above the crack as well. Above the surface, we apply no-stress conditions on the side boundaries and then drive the top of the extended domain at a prescribed velocity $u_{x,max}$, and with no vertical stress (assume a continuous fluid above). Horizontally away from the crack, the solution returns to simple shearing flow with no-slip at the amphibole and a parabolic increase in velocity towards a height A above the amphibole. The numerical solution is computed using staggered finite differences on a 5-point stencil in 2D for a linearly compressible fluid with a compressibility of 10^{-10}, using a Newton-Raphson iterative scheme. We assume there is no velocity into the plane. The numerical derivatives were calculated using a 3-point centered stencil, and 2-point stencils on the domain boundaries.

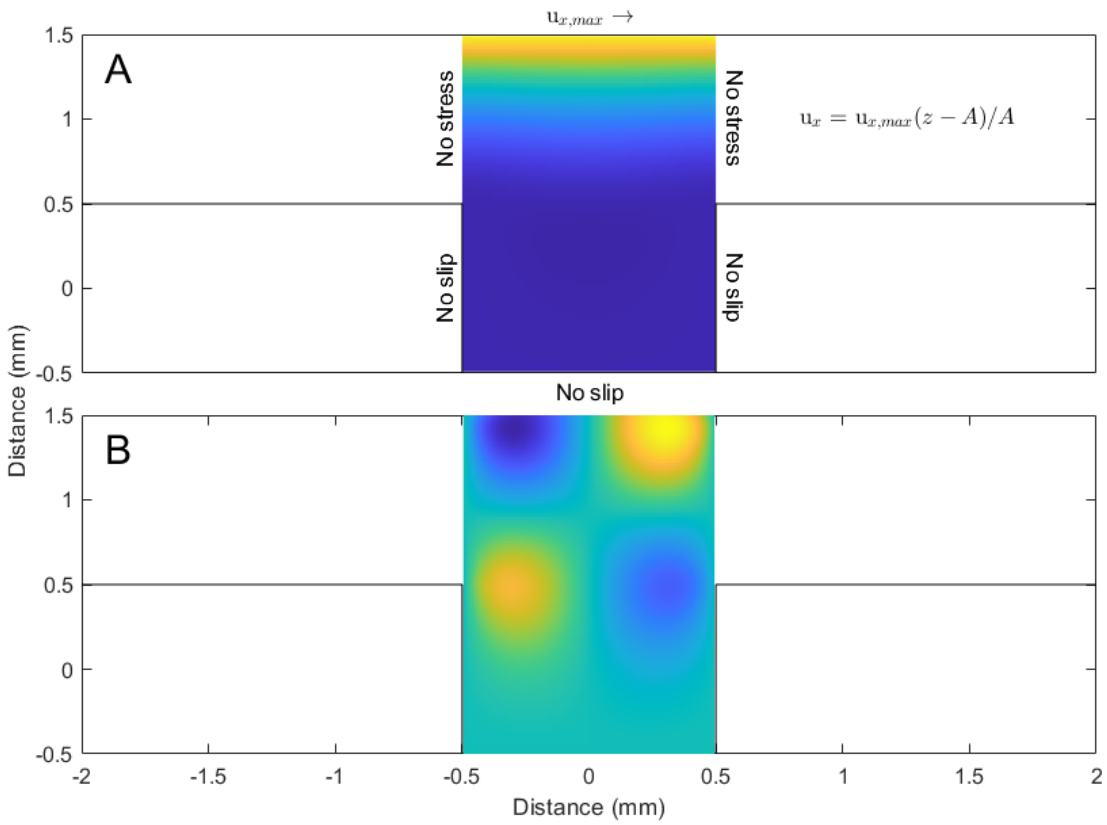

Supplementary Fig. 18*: Boundary conditions and numerical solutions for velocity (A) and strain rate (B) for the modified lid-driven cavity showing circulation flow within the crack and shearing flow above.*

We initialize 100 clinopyroxenes within the cavity and 300 randomly placed above the amphibole surface. Their aspect ratios are the same as in the gravitational settling geometry.

Simple shear flow

The general case of the velocity field surrounding a rotating ellipsoid was solved by Jeffery[7]. In the simplified case of a sphere the velocity field is:

$$u_x = \frac{-21xyzA^5(x^2+y^2+z^2-A^2)}{4(x^2+y^2+z^2)^{7/2}} \tag{8}$$

$$u_y = \frac{-21y^2zA^5(x^2+y^2+z^2-A^2)}{4(x^2+y^2+z^2)^{7/2}} - \frac{zA^7}{2(x^2+y^2+z^2)^{7/2}} \tag{9}$$

$$u_z = \frac{-21yz^2A^5(x^2+y^2+z^2-A^2)}{4(x^2+y^2+z^2)^{7/2}} + y\left(1 - \frac{A^7}{2(x^2+y^2+z^2)^{7/2}}\right) \tag{10}$$

for shear in the yz-plane.

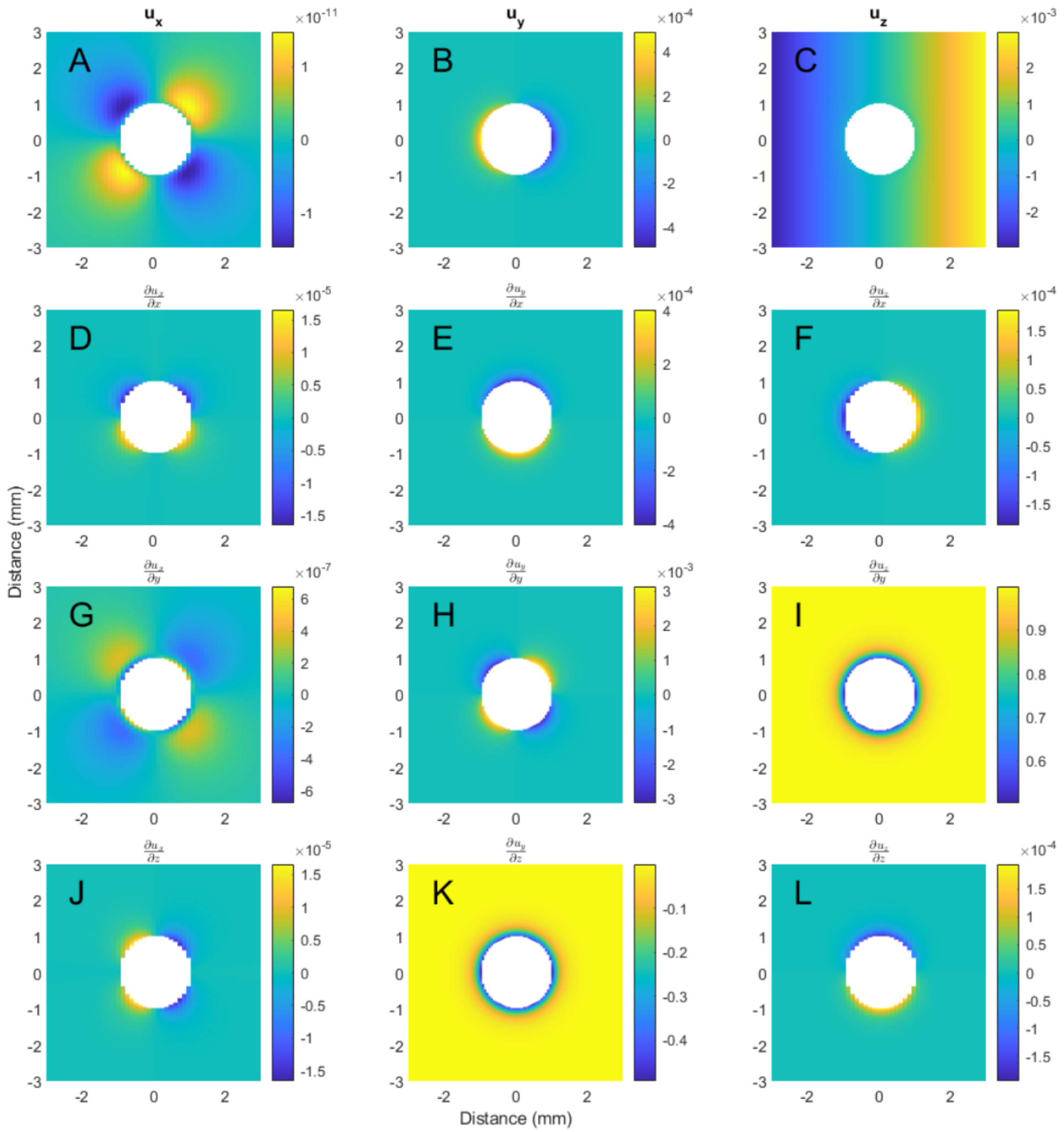

***Supplementary Fig. 19**: Analytical solutions for velocity (A-C) and strain rates (B-L) surrounding a sphere rotating in pure shear with a characteristic velocity of ±3 mm/s.*

The initial 2× 10^4 clinopyroxenes are distributed normally about 1.005A.

Calculation of crystal orientations

The analytical solutions for particle rotation require a rotation into a frame of reference aligned with the direction of maximum shear. From the strain rate tensor, we solve the Eigenvector problem to find the principal stresses at each clinopyroxene location, and choose the maximum shear to be 45° to the maximum and minimum compressive stresses. For the analytical solutions for flow fields 1&3, the strain tensor is calculated directly at the clinopyroxene centers, for flow field 2 we use 2D linear interpolation.

The azimuth φ is measured from perpendicular to the maximum shear direction and in the plane of maximum shear, while the angle θ is the dip with respect to this plane. We calculate these angles from the Eigenvector for the maximum compressive strain $\vec{v}_{max}$, the intermediate compressive stress (out-of-plane of maximum shear), $\vec{v}_{mid}$, and the unit-vector that defines the direction of the clinopyroxene c-axis, $\vec{c}$:

$$\theta = \frac{\pi}{2} - \cos^{-1}(\vec{v}_{med} \cdot \vec{c}) \tag{11}$$

And the projection of the clinopyroxene c-axis into the plane of maximum shear:

$$\vec{c}_{\phi} = \vec{c} - (\vec{v}_{med} \cdot \vec{c})\vec{v}_{med} \tag{12}$$

$$\phi = \frac{\pi}{4} + \cos^{-1}\left(\frac{\vec{v}_{max} \cdot \vec{c}_{\phi}}{|\vec{c}_{\phi}|}\right) \tag{13}$$

At which point the azimuth (ϕ') and dip (θ') after an incremental amount of simple shear strain, γ, and pure shear strain, ε, become:

$$\phi' = \phi + \gamma \frac{R^2 \cos^2 \phi + \sin^2 \phi}{R^2 + 1} + \varepsilon \frac{R^2 - 1}{(R^2 + 1)} \sin 2\phi \tag{14}$$

$$\tan \theta' = \tan \theta \frac{R^2 \cos^2 \phi + \sin^2 \phi}{R^2 \cos^2 \phi' + \sin^2 \phi'} \tag{15}$$

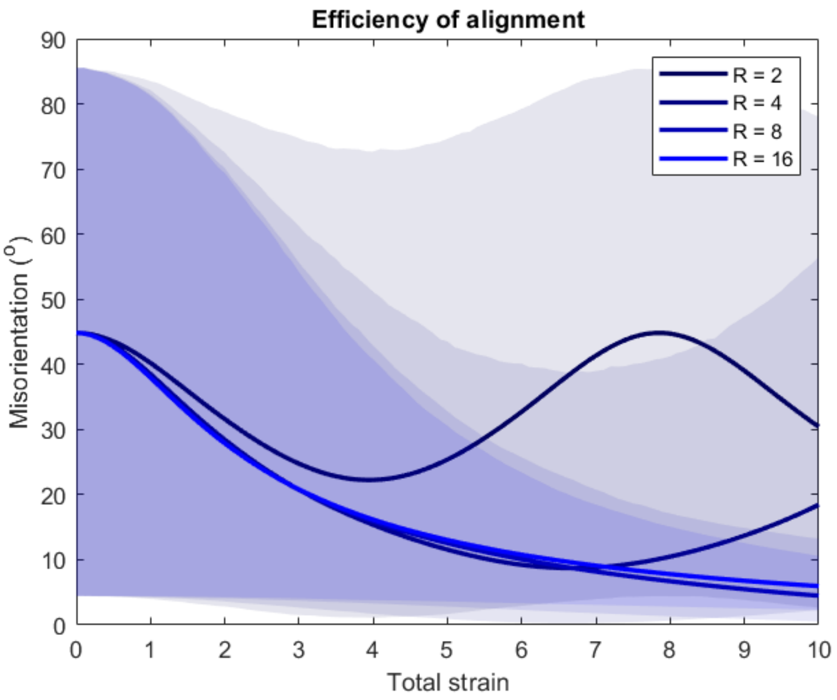

Supplementary Fig. 20*: Example calculations for a population of randomly oriented ellipsoids with different aspect ratio (R) aligning under a simple shear field. Low aspect ratio particles (R=2) show evidence of tumbling (periodic solution with long decay times), while high aspect ratio particles rapidly align to the flow field.*

And the positions of the clinopyroxene centers are updated according to:

$$x' = x + u_x \Delta t \tag{16}$$

$$y' = y + u_y \Delta t \tag{17}$$

$$z' = z + u_z \Delta t \tag{18}$$

The orientations of the clinopyroxenes are transformed back to native coordinates and their misorientation from the host amphibole is calculated using the dot product between the updated clinopyroxene direction and the amphibole direction.

We can then choose planes of intersection that cut the amphibole and halo of clinopyroxenes along a plane that need not be aligned with the amphibole or pass through the amphibole center. We specify planes of interest and select only those clinopyroxenes whose centers fall within a threshold distance from the plane, which we choose to be $A/50$ for convenience.

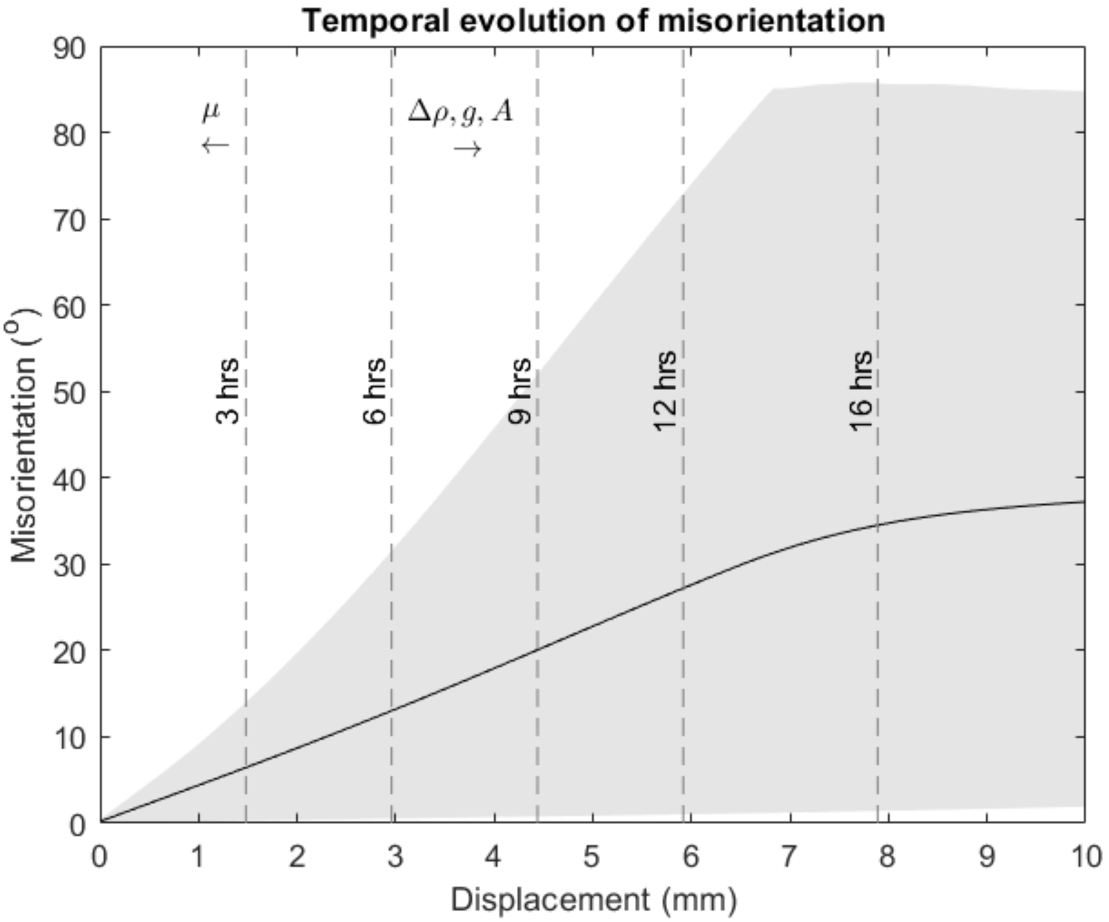

Supplementary Fig. 21: *Example calculation of evolution of misorientation for initially aligned clinopyroxenes during gravitational settling. The displacement required to reorient clinopyroxenes to the flow field is a function of the amphibole size and clinopyroxene aspect ratio (smaller amphiboles and larger clinopyroxene aspect ratio decrease required displacement). The required time depends on the settling velocity, which is itself a function of melt viscosity, the density contrast between the amphibole and the melt, gravitational acceleration, and amphibole size. The characteristic time for realignment decreases with decreasing viscosity or increasing density contrast, gravity, and amphibole size.*

Assembling mixed populations of crystals

We then investigate a conceptual model in which shear acts upon the crystals during on-going crystal nucleation. In this case, pyroxene crystals that nucleate early in the history of amphibole breakdown will see greater accumulated strains than crystals that nucleate later. We discretize this problem into ten separate time windows and nucleate a new number of crystals, n_p, in each. At every new time step, the crystals nucleated in every previous timestep are progressed by the same amount of total, far-field shear, $\Delta\varepsilon$. Numerically, this is equivalent to initializing ten separate populations, shearing each to the appropriate total strain for that population, and then adding together their misorientation distributions together in appropriate (not necessarily equal) proportions.

For illustration, we consider two end-member scenarios: 1) the rate of crystal nucleation (the number of new crystals in each time step) is held constant, but the shear rate increases or decreases with time, and 2) the shear rate is held constant, but the nucleation rate changes with time. If we consider the pyroxene nucleation rate:

$$G = G_0 e^{t/\tau_G} \tag{19}$$

and the shear strain rate:

$$\dot{\varepsilon} = \dot{\varepsilon}_0 e^{t/\tau_{\dot{\varepsilon}}} \tag{20}$$

We can quantify the competition of these two effects by comparing the relative rates of nucleation vs shear rate change, which yields a Damköhler number:

$$Da = \ln\left(\frac{e^{\frac{1}{\tau_G}}}{e^{\frac{1}{\tau_{\dot{\varepsilon}}}}}\right) = \frac{1}{\tau_G} - \frac{1}{\tau_{\dot{\varepsilon}}} \tag{21}$$

and we retain the sign of the time scale to indicate whether the rate is increasing or decreasing. Due to the linearity above, these two processes have the same effect in which, for example, exponentially decreasing strain rate or increasing nucleation rate with time, both produce a greater number of pyroxene crystals that have seen little accumulated strain, and therefore retain a stronger topotactic relationship. The reverse situation of increasing strain rate or decreasing nucleation rate produce a greater proportion of highly misaligned pyroxenes.

Supplementary Table 3: *Model parameters for crystal settling, lid-driven cavity and simple shear simulations.*

INPUT PARAMETERS	VALUE		
	Settling	Lid-driven cavity	Simple shear
AMPHIBOLE RADIUS	$5x10^{-4}$ m	>>$1x10^{-3}$ m	$1x10^{-3}$ m
VELOCITY	$1x10^{-8}$ m/s	$1x10^{-3}$ m/s	$1x10^{-3}$ m/s
TIME	48 hr	50 s	100 s
STRAIN	3.7	50	100
NUMBER OF PYROXENES PER ORIENTATION	1000	400	1000
NUMBER OF ORIENTATIONS	100	1	100
RIM THICKNESS	$1.5x10^{-5}$ m	$1x10^{-3}$ m	$3x10^{-5}$ m
ASPECT RATIO CENTER	8	8	8
ASPECT RATIO STD	5	5	5

Inversion of natural samples

We simulate ascent by assuming that the magma moves through each discrete pressure step (of 0.1 MPa) in some duration Δt, during which time it nucleates n_p pyroxene crystals at a rate proportional to the Gibbs free energy of the breakdown reaction.

We calculate the Gibbs free energy of formation of the clinopyroxene, orthopyroxene, plagioclase, and melt phases using an isothermal decompression path from 200 MPa at 860 °C using Rhyolite-MELTS[8], the amphibole after Ogorodova et al.[9], Holland[10] and Robie and Bethke[11] for the amphibole composition of Buckley et al.[12], and iron-titanium oxides (magnetite structure) after Ryabukhin et al.[13], and water after Cox et al.[14] and Chase[15]. We use the mass balance from Buckley et al.[12] with an additional fraction of melt being consumed during breakdown.

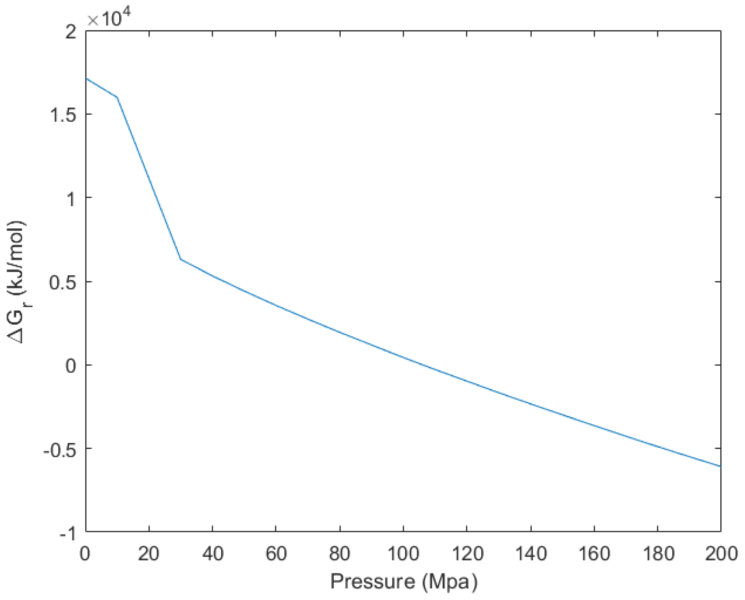

***Supplementary Fig. 22**: Gibbs free energy of the amphibole breakdown reaction as a function of pressure.*

Each population than undergoes a shear strain in its ascent to the surface, $\varepsilon = \frac{fP_n}{3R\rho g}$, where P_n is the pressure of nucleation, R is the conduit radius, which we assume to be a constant 250 m, ρ is the density of the host rock, which we assume to be a constant 2700 kg/m^3, g is the acceleration due to gravity, 9.81 m/s^2, and f is a geometrical factor that reduces the effective shear strain to account for localization at the conduit margins, which we set to 1/10.

The final population of misoriented crystals is a summation over each sub-population associated with each pressure. We produce template populations for each pressure (shear strain) using the conditions from section 1.3 for 100 different initial amphibole orientations with 1000 pyroxenes each. We consider the full 3D distribution to account for the arbitrary sampling surfaces. These are the same populations shown in Fig. 6 of the main text.

For our case example, we take four samples from Soufrière Hills Volcano which have between 5 and 20 amphiboles each, representing between 2347 and 27912 pyroxene orientations. We perform a random walk Markov Chain Monte Carlo (MCMC) inversion with 1000 walkers for 500 steps, initially at half resolution in pressure to improve convergence speed and reduce high-frequency fluctuations between adjacent populations with similar populations. We take the best result from the coarse fit and use a spline interpolation to double the resolution and initialize 1000 additional walkers which also iterate over 500 steps. This entire process was performed 5 times and the best result for each sample was chosen from these 5 posterior distributions.

The MCMC inversion results produce a normalized time at each pressure (depth), but not an absolute value. We use existing estimates for the rate of amphibole rim growth from Rutherford and Hill[16], Hammer and Rutherford[17], Rutherford and Devine[18], Browne and Gardner[19], and De Angelis et al.[4] to construct reasonable crystal growth rates at the corresponding pressures and temperatures for this composition. We arrive at a simplified bi-linear fit which approximates both the increasing growth rate with increasing degree of disequilibrium at high pressure and the decreasing growth rate at lower pressures where the melt viscosity becomes high and the diffusivity is low. We neglect the effect of time on growth rates and assume that the single-step decompression experiments with durations 1–5 days best represent the likely decompression time scales for the Soufrière Hills Volcano eruptions. We then scale the total time from our inversion results such that the integral overgrowth at each time step is the final measured rim thicknesses from our samples. Specifically, growth rates are parameterised using a linear interpolation between fixed pressure–growth-rate anchor points: 300 MPa (0 μm day^{-1}), 110 MPa (0 μm day^{-1}), 105 MPa (7 μm day^{-1}), and 0 MPa (0 μm day^{-1}). These values define the pressure-dependent growth-rate function used to scale the normalised MCMC time output into absolute time and decompression rates.

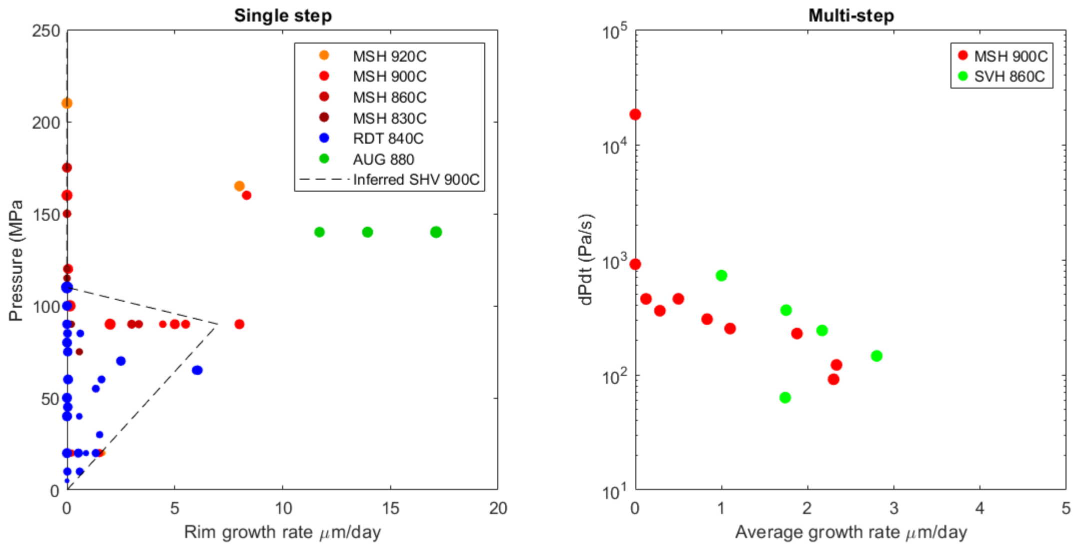

Supplementary Fig. 23*: Summary of available data for rim growth with pressure based on experimental data from Mount St. Helens (MSH)[16], Redout volcano (RDT)[19], and Augustine volcano (AUG)[4], and decompression rate from MSH and Soufrière Hills Volcano (SHV)[18], along with our inferred growth rates for SHV at 900 °C.*

Supplementary References

1. Turner, S. J., Izbekov, P. & Langmuir, C. The magma plumbing system of Bezymianny Volcano: Insights from a 54 year time series of trace element whole-rock geochemistry and amphibole compositions. *J. Volcanol. Geotherm. Res.* **263**, 108–121 (2013).
2. Tepley, F. J., De Silva, S. & Salas, G. Magma dynamics and petrological evolution leading to thevei 5 2000 BP Eruption of EL mistivolcano, southern Peru. *J. Petrol.* **54**, 2033–2065 (2013).
3. Wallace, P. A. *et al.* Petrological architecture of a magmatic shear zone: A multidisciplinary investigation of strain localisation during magma ascent at Unzen volcano, Japan. *J. Petrol.* **60**, 791–826 (2019).
4. De Angelis, S. H., Larsen, J., Coombs, M., Dunn, A. & Hayden, L. Amphibole reaction rims as a record of pre-eruptive magmatic heating: An experimental approach. *Earth Planet. Sci. Lett.* **426**, 235–245 (2015).
5. Wallace, P. A. *et al.* CO2 flushing and redox as drivers of pre-eruptive amphibole breakdown. *Earth Planet. Sci. Lett.* **667**, 119532 (2025).
6. Stokes, G. G. On the effect of the internal friction of fluids on the motion of pendulums. *Transactions of the Cambridge Philosophical Society* **9**, 8 (1851).
7. Jeffery, G. B. The motion of ellipsoidal particles immersed in a viscous fluid. *Proc. R. Soc. Lond. A Math. Phys. Sci.* **102**, 161–179 (1922).
8. Gualda, G. A. R., Ghiorso, M. S., Lemons, R. V. & Carley, T. L. Rhyolite-MELTS: A modified calibration of MELTS optimized for silica-rich, fluid-bearing magmatic systems. *J. petrol.* **53**, 875–890 (2012).
9. Ogorodova, L. P. *et al.* Thermodynamic study of calcic amphiboles. *Geochem. Int.* **55**, 814–821 (2017).
10. Holland, T. J. B. Dependence of entropy on volume for silicate and oxide minerals: A review and a predictive model. *Am. Mineral.* **74(1–2)**, 5–13 (1989).

11. Robie, R. A. & Bethke, P. M. *Trace Elements Investigations: Molar Volumes and Densities of Minerals*. https://pubs.usgs.gov/tei/822/report.pdf (1962).

12. Buckley, V. J. E., Sparks, R. S. J. & Wood, B. J. Hornblende dehydration reactions during magma ascent at Soufrière Hills Volcano, Montserrat. *Contrib. Mineral. Petrol.* **151**, 121–140 (2006).

13. Ryabukhin, A. G., Roshin, A. V. & Roshin, V. E. Entropy of the crystalline titanomagnetites (FeO) x · TiO2. *Russ. Metall. (Met.)* **2006**, 492–495 (2006).

14. Cox, J., Wagman, D. & Medvedev, V. *CODATA Key Values for Thermodynamics*. (Hemisphere Publishing Corp., New York, 1984).

15. Chase, M. W. *NIST-JANAF Thermochemical Tables*. (American Institute of Physics, New York, NY, 1998).

16. Rutherford, M. J. & Hill, P. M. Magma Ascent Rates From Amphibole Breakdown: An Experimental Study Applied to the 1980-1986 Mount St. Helens Eruptions. *J. Geophys. Res.* **98**, (1993).

17. Hammer, J. E. & Rutherford, M. J. An experimental study of the kinetics of decompression-induced crystallization in silicic melt. *J. Geophys. Res. [Solid Earth]* **107**, ECV 8-1-ECV 8-24 (2002).

18. Rutherford, M. J. & Devine, J. D. Magmatic conditions and magma ascent as indicated by hornblende phase equilibria and reactions in the 1995-2002 Soufrière Hills magma. *J. Petrol.* **44**, 1433–1454 (2003).

19. Browne, B. L. & Gardner, J. E. The influence of magma ascent path on the texture, mineralogy, and formation of hornblende reaction rims. *Earth Planet. Sci. Lett.* **246**, 161–176 (2006).
